# Nanoparticle-Based Therapeutic Approach for Diabetic Wound Healing

**DOI:** 10.3390/nano10061234

**Published:** 2020-06-25

**Authors:** Hariharan Ezhilarasu, Dinesh Vishalli, S. Thameem Dheen, Boon-Huat Bay, Dinesh Kumar Srinivasan

**Affiliations:** 1 Department of Anatomy, Yong Loo Lin School of Medicine, National University of Singapore, Singapore 117594, Singapore; anthe@nus.edu.sg (H.E.); antstd@nus.edu.sg (S.T.D.); antbaybh@nus.edu.sg (B.-H.B.); 2 Faculty of Medical Sciences, Krishna Institute of Medical Sciences “Deemed to be University”, Karad, Maharashtra 415539, India; vishallidinesh@gmail.com

**Keywords:** nanoparticle, drug delivery system, diabetes mellitus, wound healing, diabetic foot ulcer, pathophysiology

## Abstract

Diabetes mellitus (DM) is a common endocrine disease characterized by a state of hyperglycemia (higher level of glucose in the blood than usual). DM and its complications can lead to diabetic foot ulcer (DFU). DFU is associated with impaired wound healing, due to inappropriate cellular and cytokines response, infection, poor vascularization, and neuropathy. Effective therapeutic strategies for the management of impaired wound could be attained through a better insight of molecular mechanism and pathophysiology of diabetic wound healing. Nanotherapeutics-based agents engineered within 1–100 nm levels, which include nanoparticles and nanoscaffolds, are recent promising treatment strategies for accelerating diabetic wound healing. Nanoparticles are smaller in size and have high surface area to volume ratio that increases the likelihood of biological interaction and penetration at wound site. They are ideal for topical delivery of drugs in a sustained manner, eliciting cell-to-cell interactions, cell proliferation, vascularization, cell signaling, and elaboration of biomolecules necessary for effective wound healing. Furthermore, nanoparticles have the ability to deliver one or more therapeutic drug molecules, such as growth factors, nucleic acids, antibiotics, and antioxidants, which can be released in a sustained manner within the target tissue. This review focuses on recent approaches in the development of nanoparticle-based therapeutics for enhancing diabetic wound healing.

## 1. Introduction

**Figure 1:**
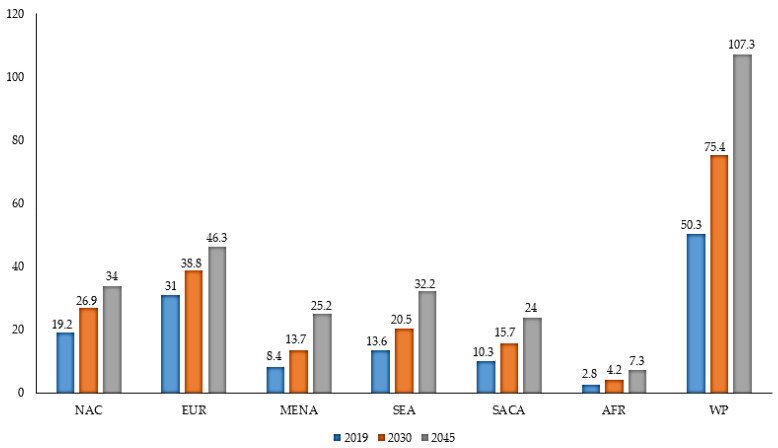
Prevalence of DM (millions) by IDF regions in adults (>65 years) in 2019, 2030 and 2045 [3]. IDF: International Diabetes Federation; NAC: North America and Caribbean; EUR: Europe; MENA: Middle East and North Africa; SEA: South-East Asia; SACA: South and Central America; AFR: Africa; WP: Western Pacific.

Diabetes mellitus (DM) is a chronic health problem that is prevalent among the human population. DM is an endocrine disorder which is distinguished by the state of hyperglycemia (higher level of glucose in the blood), and is classified into Type 1 DM and Type 2 DM. Factors associated with a steady increase in DM are aging populations, dietetic revolutions and sedentary lifestyles [1,2]. On the basis of 2019 prevalence data from the International Diabetes Federation (IDF), the estimated number of adults (20–79) with DM worldwide is 463 million, which is expected to increase to 578.4 million by 2030 and 700.2 million by 2045 [3]. It is anticipated that DM may increase in developing countries as compared to developed countries (Figure 1). In 2019, IDF revealed that the number of deaths resulting from DM and its complications was 4.2 million worldwide [3]. It is projected that the annual global health expenditure on DM in 2019 is USD 760 billion, which will reach USD 825 billion by 2030 and USD 845 billion by 2045 [3]. Therefore, DM has emerged as one of the serious health threats with a huge socioeconomic burden.

DM increases the risk of infection and delays wound healing due to impairment of metabolic activity. As DM advances, a complication that may occur is diabetic foot ulcers (DFUs), a chronic wound that affects the lifestyle of patients and consequently, heightening the risk of mortality [1]. Worldwide, 9.1 to 26.1 million people with DM develop DFU annually. Individuals with DM stand a 25% chance of risk for DFU, and sadly, many cases must ultimately opt for amputation as the treatment modality. Fifty percent DFU amputees have an average 3-year survival rate as a result of infection and unsolved arterial injury, while for post-treatment patients with healed DFU, 50% to 70% may have recurrence within 5 years [1,4,5]. Though DFU is preventable, it puts a massive burden on patients and health care services. A cautious lifestyle as a preventive front, timely assessment and high-level treatments by a multi-disciplinary group of specialists are effective approaches for DFU management [6].

### 1.1. Pathophysiology of Diabetic Foot Ulcer (DFU)

**Figure 2:**
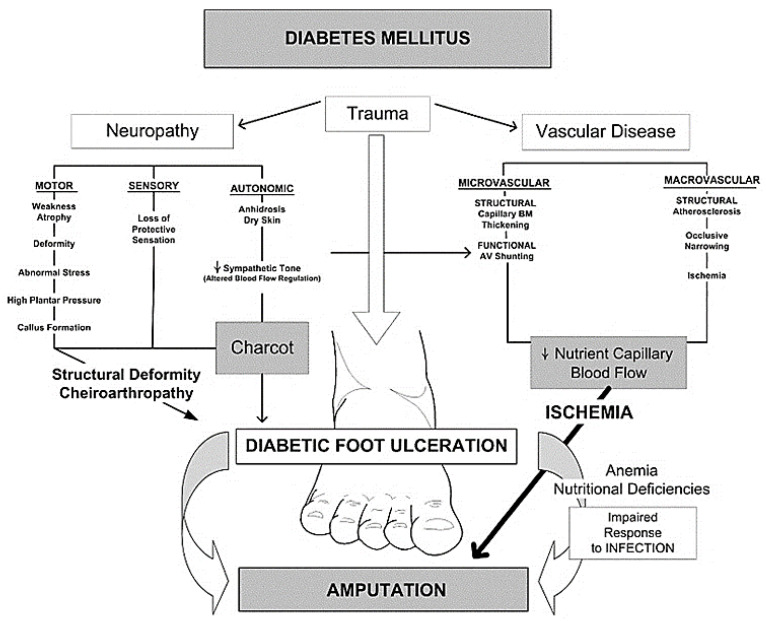
Pathophysiology of DFU. Reproduced from [7], with permission from Elsevier, 2006.

Peripheral arterial disease (PAD), neuropathy, ischemia, and infection are the key factors influencing the development of DFU. Figure 2 shows a flow diagram depicting the factors that contribute to the pathophysiology of DFU [7].

### 1.2. Neuropathy

DFU may develop as a result of neuropathy caused by hyperglycemia [8]. The hyperglycemic condition increases stimulation of the enzymes, aldose reductase and sorbitol dehydrogenase, which lead to conversion of intracellular glucose to sorbitol and fructose. The accumulation of converted glucose products results in a decrease in the synthesis of nerve cell myoinositol [9]. In addition, the chemical change associated with glucose induces depletion of nicotinamide adenine dinucleotide phosphate (NADP), which is essential for the detoxification of reactive oxygen species (ROS) and for the synthesis of the vasodilator, nitric oxide (NO). There is a subsequent upsurge in oxidative stress on the nerve cells and an increase in vasoconstriction leading to ischemia, which will cause nerve cell damage and cell death [10,11]. Neuropathy affects all the components of the nervous system, viz., sensory, motor and autonomic. In autonomic neuropathy, the foot becomes dry as it loses the ability to moisturize its surface due to decreased secretory functions of the sebaceous and sweat glands, thereby encouraging infections to spread [5,8].

### 1.3. Peripheral Arterial Disease (PAD)

DFUs are also known to be caused by the complications of PAD. Multiple factors other than DM are associated with greater risk of PAD including age, smoking, hypertension, hyperlipidemia, inflammatory markers, and renal dysfunction [12]. Diabetic vascular complications are divided into microvascular and macrovascular disease. In the diabetic state, due to the upsurge in glucose, endothelial cellular dysfunction and smooth muscle abnormalities develop as a consequence of a reduction in endothelium-derived vasodilators, leading to constriction of blood arteries in the foot [13]. Furthermore, atherosclerosis with thickening of blood capillaries and hardening of arteriolar walls, cause blockage in major arteries such as femoro-popliteal and aortoiliac vessels, resulting in ischemia [2].

## 2. Normal and Diabetic Wound Healing

**Figure 3:**
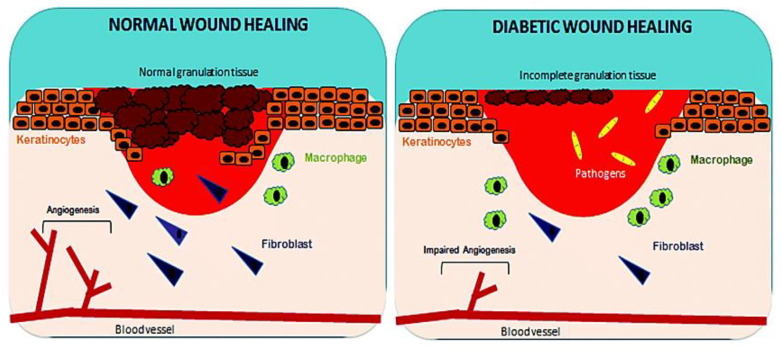
Factors affecting normal and diabetic wound healing. Reproduced from [1], with permission from Royal Society of Chemistry, 2017.

Wound healing is a complex process with dynamic interactions of different cell types, extracellular matrix (ECM), cytokines and growth factors. The fundamental steps of wound healing include hemostasis, inflammation, cell movement, and proliferation, followed by wound compression and further remodeling [14]. Any bleeding associated with penetration of skin to the dermis layer by trauma is considered as a wound [15]. The first step in initiating the wound healing process is hemostasis, a clotting process involving the coagulation cascade that leads to cessation of bleeding. The first subset of cells that enter the injury site are platelets, which release several growth factors such as platelet derived growth factor (PDGF), transforming growth factor beta (TGF-β), endothelial growth factor (EGF), and fibroblast growth factor (FGF), which support the inflammation process [16,17]. The inflammatory phase occurs immediately after hemostasis and is characterized by vascular delivery of inflammatory agents and migration of cells into the injury site. Release of inflammatory mediators, such as prostaglandins, histamine and leukotrienes by mast cells, which stimulates angiogenesis and permeability to allow cells and molecules from the blood stream to enter the wound site [18,19]. Neutrophils, monocytes and lymphocytes are white blood cells that invade the injury site. Neutrophils combat microbial infections and macrophages, stimulate angiogenesis by secretion of TGF-β, vascular endothelial growth factor (VEGF) and FGF, and produce tumor necrosis factor alpha (TNF-α), which breakdown necrotic tissue, facilitating the proliferation of fibroblasts that deposit collagen for tissue granulation [20,21]. Wound contraction begins 2 weeks after a dermal wound. During tissue granulation, fibroblasts differentiates to myofibroblasts phenotype, with enhanced alpha smooth muscle actin (α-SMA) cytoskeleton, which plays a vital role in wound closure. Re-epithelialization of tissue occurs when the wound bed is covered by new tissue and keratinocytes migrate, differentiate and proliferate to generate a stratified epidermis along the superficial area of injury, providing cover for newly formed tissue [22,23]. The last phase in the wound healing process (which lasts 6 to 24 months) is wound remodeling. In this phase, granulation tissue forms accompanied by replacement of the ECM with type I collagen (substituting collagen III) mediated via PDGF and TGF-β [24,25] (Figure 3).

In diabetic wounds, a larger number of inflammatory macrophages continue to stay at the site of injury for a longer period, compared to normal wound healing. These macrophages produce an increased ratio of pro-inflammatory cytokines, such as TNF-α and interleukin 6 (IL-6) and elaborate ROS causing persistent inflammation, which lead to stimulation of proliferative factors for successful wound healing. However, the common cytokine cascade is perturbed due to inefficient efferocytosis (phagocytosis of apoptotic cells) by macrophages, related to the higher burden of apoptotic cells. Increased ratio of pro-inflammatory cytokines (IL-1β and TNF-α) and matrix metalloproteinase-9 (MMP-9) with decreased anti-inflammatory signals (CD206, IGF-1, TGF-β and IL-10) will lead to abnormal apoptosis of fibroblasts and keratinocytes, together with decreased angiogenesis [26,27,28]. In diabetic wound healing, fibroblasts do not properly differentiate into myofibroblasts, leading to reduced mechanical tension of ECM, and subsequently poor wound closure due to lack of α-SMA [28,29,30]. In impaired wound healing, a non-equilibrium balance between MMPs that degrade the disorganized collagen in normal wound healing and tissue inhibitor of metalloproteinases (TIMPs), lead to abnormal ECM degradation and deposition. Lower expression of TIMPs and higher expression of MMPs are due to the persistently high levels of pro-inflammatory cytokines and pro-fibrotic cytokines. In a chronic wound, levels of MMPs are raised 60 times more than that for acute wound healing [30]. Increase in protease activity in tissue reconstruction enhances degradation of ECM, growth factors and collagen deposition, which are crucial for effective wound healing [31,32]. All these factors, together with a dysregulated molecular and cellular wound microenvironment that is not conducive to normal healing responses, culminate in impaired healing of diabetic ulcer [1] (as illustrated in Figure 3).

## 3. Therapeutic Modalities for Diabetic Foot Ulcers

Chronic wounds remain a significant public health problem. Alterations in normal physiological healing processes caused by aging or diabetes, lead to impaired tissue repair and the development of chronic and non-healing wounds. Understanding the unique features of the wound environment will be required to develop new therapeutics that impact these disabling conditions. Although there are numerous strategies for the treatment of DFU, it remains a major challenge to optimize the therapeutic approach in the clinical healthcare setting [33]. Systemic delivery, the most common approach for administering drugs to patients, relies on adequate perfusion of the target tissue and blood supply, that many chronic wounds lack. Moreover, there may be significant potential harmful side-effects to non-target tissues. On the other hand, topical delivery is primarily intended for a local effect which can potentially eliminate the need for systemic administration of drug therapies, minimize the total dose required to reach the target site, and reduce off-target adverse effects [34,35]. Wound care has traditionally relied on dressings, including both natural and synthetic materials and drugs, to sustain a warm and moist surrounding for conducive wound healing, while diminishing bacterial infection [35]. Topical delivery of free siRNA, proteins, antibiotics, and nucleic acids, may lead to degradation of these encapsulated compounds by endogenous enzymes produced in chronic wounds, increased drug clearance due to rapid half-life, toxicity to tissues or organs, and uncontrolled delivery of drugs, leading to under dosage or over dosage and inappropriate immune responses [36,37]. Moreover, topical application of therapeutic drugs offers a poor solution with regard to diabetic wound healing due to the development of bacterial resistance against antibiotics [38].

Other diabetic wound healing therapies, such as bioengineered grafts, face the problems of decreased angiogenesis and physiological rejection. Growth factor therapy may encounter problems associated with breakdown of growth factor at wound site, synthetic hydrophobic polymer dressings with ineffective release of bioactive components, silver dressings with cellular toxicity at specific concentrations, and natural polymer dressings may give rise to allergic reactions [39]. Commercially available hydrofiber and hydropolymer dressings, as well as alginates, are not suitable for dry wounds. On the other hand, hydrocolloidal dressings require a secondary dressing to prevent contamination and also not an option for substantial draining wounds. Foam dressings may cause dehydration of wounds, which arrest epithelialization of the ulcer. Currently, there is no experimental verification of a single type of wound dressing that is effective in eliminating every limitation posed by DFU [40]. 

## 4. Nanotechnology Based Drug Delivery System

New drug-delivery systems (DDSs) may enhance the current and future therapies for this challenging clinical problems [35]. Recently, nanotechnology has become one of the most focused research areas for the treatment of DM patients and its associated complications. The advantage of nanomaterials (with a range of 1–100 nm) are versatility in use, controlled size, and tunability of physiochemical properties. Nanomaterials with a larger surface area to volume ratio allow for cell adhesion, and possibly can encapsulate a greater number of surface functionalized active components to accelerate specific regenerative functions [41]. The nanotechnology-based wound healing methods confer advantages such as topical drug delivery, cell specificity, and sustainable and controlled release of encapsulated drugs for a required period until the wound heals [34,42]. In the case of wound healing, nanoparticles are ideal for topical delivery, supporting better interactions with the biological target and increased penetration at the wound sites. Besides, encapsulated drugs could be delivered in a sustained manner and delivery rate could be suitably altered by changing the nanoparticle distribution. Thus, wound healing treatments incorporating the nanotherapeutics approach for delivery of therapeutic biomolecules, paves the way for an excellent opportunity to tackle the complexity of diabetic wound healing by [43,44]. 

**Figure 4:**
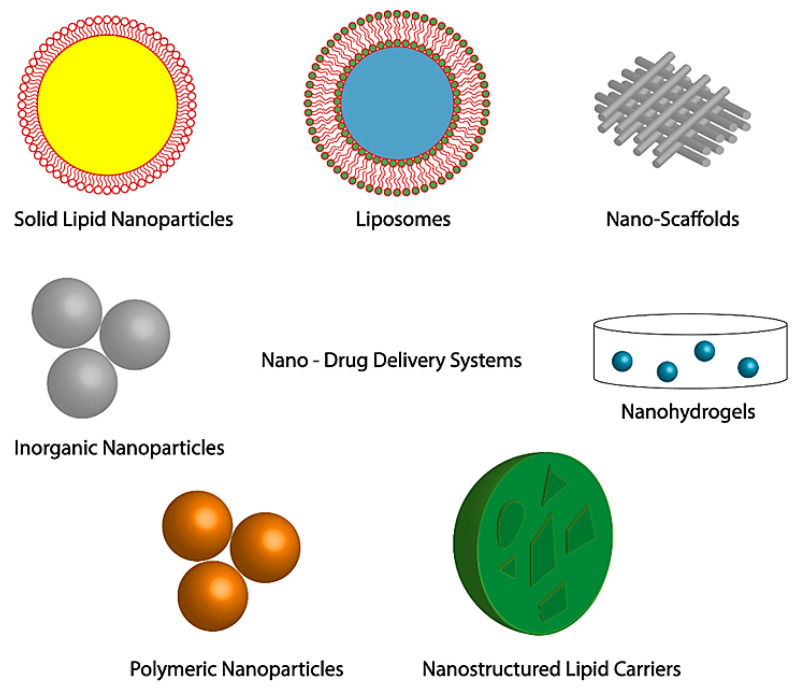
Nano-DDSs in skin regeneration and wound treatment. Adapted from [46], with permission from Springer Nature, 2019.

Conceptually, topical delivery of nanotherapeutics has major advantages for chronic wounds such as diabetic wound, by promoting effective wound healing and skin regeneration due to: (a) multifactorial factors and cell-type specificity and (b) use of therapeutic agent for a limited time or until the wound has healed. Nanotechnology-based materials act as smart nanomaterials in the form of nanofibers and hydrogel, foams loaded with nanoparticles which can encapsulate antibiotics, growth factors, peptides, nucleic acids and extracellular substrates, with the possibility of combined delivery of two different therapeutic agent with dissimilar characteristics to enhance the healing process, that include liposomes, polymeric nanoparticles, inorganic nanoparticles, lipid nanoparticles, nanofibrous structures, and nanohydrogel [34,39,45,46,47] (as shown in Figure 4). 

**Figure 5:**
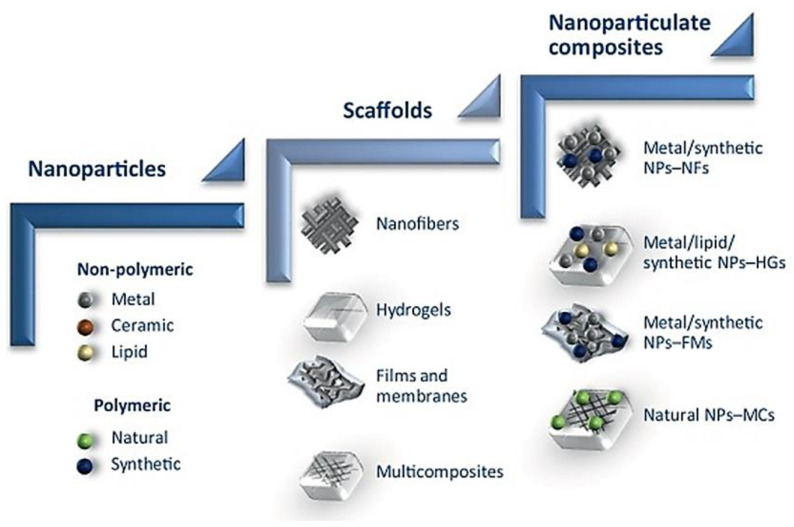
Schematic of nanomaterial, from nanoparticle to nanoparticulate system for wound regeneration. NFs—Nanofibers, NPs—Nanoparticles, HGs—Hydrogel, FMs—Films and membrane, MCs—Multicomposites. Reproduced from [49], with permission from Elsevier, 2017.

Drugs are absorbed, dispersed or dissolved around the nanoparticle, and confined in an aqueous core with shell like surroundings, or alternatively the drug can be covalently bound to the surface matrix of nanoparticles [48]. In the biological system, the drugs loaded in the nanoparticles will be released by diffusion, dissolution, reduction and distension. Furthermore, nanoparticles can be encapsulated in nanofiber, hydrogel, foam, films and nanocrystals as a nanocomposite system along with other drugs (Figure 5), which allows for synergistic effect between nanoparticles and the drug of interest, creating a new concept of wound dressing that promotes enhanced wound healing [49]. Such dressings have increased porosity surface-to-volume ratio, and their structure simulates the topographic appearance of endogenous ECM, allowing attachment and spreading of both fibroblasts and keratinocytes, thereby facilitating collagen synthesis and re-epithelialization of wounds [41].

## 5. Nanoparticle Delivery of Therapeutic Drugs for Diabetic Wound Healing

It is well established that delivering of therapeutic active components such as growth factors, nitric oxide, nucleic acid, antioxidants, and antibiotics to damaged tissue, can stimulate cell proliferation, migration, angiogenesis, and collagen secretion, and inhibit microbes, thereby influencing healing of chronic wounds [50]. Nanofibers have received much attention because of their structural similarity, which closely mimics the native ECM environment [51,52]. Nanofibers promote wound healing by providing characteristics of high surface area to volume ratio, tunable mechanical properties, increased porosity, and ability to encapsulate nanoparticles and bioactive compounds for controlled release, which can support the cells to actively interact with the matrix during functionalization and remodeling [53,54]. Hydrogels are hydrophilic 3D polymer networks with established applications in tissue engineering and drug delivery. Hydrogels with high water content, tunable viscoelasticity and biocompatibility have been intensively explored to enable topical delivery of bioactive molecules [55,56]. More importantly, nanoparticle and biomolecules can be incorporated in hydrogels and thus, opens the door to more advanced topical drug delivery with unique benefits such as improved tissue localization, minimized burst release and controlled sequential drug release, by preserving its structural integrity of nanoparticle [57]. Non-polymeric nanoparticles such as silver nanoparticles (AgNPs) and gold nanoparticles (AuNPs) are widely used as therapeutic agents, primarily for their anti-infective and anti-inflammatory effects [58]. There is an unmet need for a novel antibiofilm approach and effective antimicrobial compounds, and silver nanotechnology-based therapeutics has captured the attention of health care providers for enhancing health care [59]. AgNPs are used in clinical practice for a wide range of treatments such as burns, chronic ulcers and diabetic wounds that have developed antibiotic resistance and hospital acquired bacterial infection. In addition to anti-inflammatory effects, AgNPs treated wounds have shown abundant collagen deposition that could accelerate wound healing [60,61]. Biocompatible AuNPs are extensively used in tissue regeneration, targeted drug delivery and wound healing. Unlike Ag nanomaterials, Au nanomaterials as a single material alone does not have any antimicrobial activity. Thus, AuNPs must be incorporated with other biomolecules to be used for effective biological functions [62,63]. Zinc (Zn) can be used for treating type 1 and type 2 DM, owing to its role in the function of >300 enzymes that are necessary to maintain metabolic homeostasis in the body. Zn reduces blood sugar levels by inhibiting glucose absorption and raising glucose absorption by skeletal muscles and adipose tissues [64]. Zinc oxide (ZnO) nanoparticles have been explored as drug delivery carriers and therapeutic approaches for human biomedical applications because of the fact of their biocompatibility [65]. ZnO nanoparticles have exhibited therapeutic activities against melanoma, diabetes, bacterial infection, and inflammation, and have shown potential for wound healing applications [66]. Ceramic nanoparticles containing inorganic components have fundamental therapeutic ability and can transport drugs to injury sites [67]. Lipid-based nanoparticles, in addition to being safe, are extensively used to deliver both hydrophilic and hydrophobic drugs. Liposomes sustain long term release of drugs by reducing the toxicity exerted by huge release of drugs via conventional administration [68]. In the case of polymeric nanoparticles, chitosan is a natural polymer to use, due to its biocompatibility and antimicrobial activity. It is possible to encapsulate a wide range of natural components such as aloe vera, vitamin E and curcumin, which have potential beneficial effects on skin wound healing [69,70]. PLGA or poly (lactic-*co*-glycolic acid), poly (*ε*-caprolactone) (PCL), poly (lactic acid) (PLA), and poly (ethylene glycol) (PEG) are synthetic polymers approved by Food and Drug Administration (FDA). Among these polymers, PLGA is considered the best biodegradable polymer due to its ability to release lactate, a degradation byproduct. PLGA nanoparticles have been reported to stimulate cell proliferation and shorten the duration of wound healing in diabetic rats and despite moderate drug loading may be a promising delivery system for growth factors [71,72].

The type of therapeutics that can be delivered by nanoparticles are given below.

### 5.1. Growth Factors

Growth factors are physiologically active proteins involved in cell proliferation, migration, differentiation, and metabolism. Physiologically, every healing process is regulated by growth factors and cytokines. Growth factors bind to a specific receptor and stimulate a series of molecular mechanisms that are essential for cell function [73]. In the wound healing process, growth factors play an important role by stimulating inflammatory response, angiogenesis, granulation of tissue, and modelling. It is well established that in a diabetic wound, the availability of growth factors will decrease due to the pathophysiology [74,75]. External administration of growth factors can be given, but proteases present in the wound bed can easily degrade these growth factors physiologically. Furthermore, the short half-life of growth factors and their reasonably large size, together with toxicity at an elevated systemic dosage, shows that conventional delivery techniques of growth factor in a free form are not appropriate to transport growth factors effectively in the wound bed. In addition, as various biomolecules are engaged in wound healing progression, sometimes it may be inadequate to utilize a single growth factor to accelerate wound closure in diabetic ulcers [76,77]. With these problems, encapsulation of growth factors in nanoparticles have been widely used to overcome the limitation of protein administration by improving the half-life, encapsulation of more than one biomolecule, and protection against degradation by proteases in the wound bed through protective characteristics of nanoparticles [78]. Nanoparticle-loaded recombinant human EGF (rhEGF) has been shown to provide faster healing of wound compared to free rhEGF administration in rats, due to the sustained release of rhEGF [79]. Nanoparticle-loaded VEGF have been observed to induce faster acceleration of wound closure in both diabetic and non-diabetic mice, as compared to PLGA nanoparticle and VEGF alone [80]. Gainza et al. fabricated rhEGF loaded solid lipid nanoparticles (SLN) and nanostructure lipid carrier (NLC) using the emulsion ultrasonication method. The same investigators showed that SLN-rhEGF and NLC-rhEGF significantly increased wound closure in diabetic mice compared to free rhEGF and alginate microspheres with rhEGF, suggesting that there is controlled release of rhEGF from lipid nanoparticle without loss of rhEGF bioactivity after encapsulation [81]. In another study, Losi et al. reported that poly(ether)urethane-polydimethylsiloxane/fibrin-based scaffold containing PLGA nanoparticles loaded with VEGF and basic fibroblast growth factor (bFGF) (scaffold/growth factor-loaded NPs) stimulated significant granulation tissue formation, collagen secretion and re-epithelialization, thereby promoting considerable increase in wound closure rate in diabetic mice, as compared to scaffold with PLGA nanoparticles without growth factors and controls. The same authors further suggested that the observed results may be due to: (i) controlled delivery of growth factor from the encapsulated nanoparticles, (ii) simultaneous delivery of more than one growth factor, and (iii) administration of growth factor protecting from enzymatic hydrolysis by encapsulating in nanoparticles [82]. In another study, chitosan-based hydrogel carrying human epidermal growth factor was conjugated with sodium carboxymethyl chitosan nanoparticles (NaCMCh-rhEGF) for controlled release of growth factor in an excision wound model on diabetic rats. The in vitro results demonstrated that the NaCMCh-rhEGF stimulated higher cell viability, thereby reducing the wound area significantly on day 15 in comparison to free rhEGF and controls [83]. Lai et al. fabricated a collagen (Col)- hyaluronic acid (HA) electrospun nanofibrous scaffold encapsulated with gelatin nanoparticles that can release multiple angiogenic growth factors such as VEGF, PDGF, bFGF, and EGF at the excision wound site. Topical application of Col-HA membrane with four kinds of growth factors (Col-Ha w/4GF) on the diabetic wound bed accelerated complete healing of excision wound in rats along with elevated collagen synthesis, re-epithelialization and vascularization compared to control animals [84]. Furthermore, Li et al. conjugated keratinocyte growth factor (KGF) with AuNPs to determine the stability and binding affinity of KGF for diabetic wound healing. The result showed that by KGF-AuNPs conjugation, KGF retained its bioactive affect at the wound site at greater stability and resistance against proteolytic degradation to promote keratinocytes proliferation and migration and generated greater binding effect to its physiological receptor than unmodified KGF. Moreover, KGF-AuNPs at wound site supported re-epithelialization and wound contraction along with elevated expression of Col-I, α-SMA and TGF-β1. These observed conditions lead to accelerated wound healing by fabricated KGF-AuNPs when compared to controls [85]. Recently, the safety and efficiency of topically administered exogenous growth factors (VEGF or bFGF) in the healing of chronic diabetic wounds were examined in clinical trials, where local administration of growth factors was proven to be well tolerated. However, the free form of exogenous growth factor administration has encountered problems such as rapid leakage from the wound bed, short biological half-life and the rapid enzymatic degradation, which makes it difficult to achieve effective concentration to treat diabetic ulcer, leading to inefficacy of the treatment [86,87]. The afore-mentioned growth factor delivery by nanoparticles (as summarized in Table 1) has also addressed the common clinical barriers, which include achieving a sustained and controlled release of biomolecule proteins, distributing concurrently more than one growth factor, and protecting the growth factors against enzymatic hydrolysis when administrated at the wound site, suggesting promising future clinical application of growth factor-loaded nanoparticles for diabetic wound healing.

### 5.2. Nucleic Acid

Nucleic acid encapsulated particulate combines gene therapy and nanotechnology to knockdown or express a specific gene for successful healing of a chronic wound [88]. Gene delivery to injury site supports expressing specific proteins which can accelerate healing of chronic wounds. For instance, VEGF for induction of angiogenesis in chronic wound has been transfected by viral vectors in diabetic patients and the effect in wound healing observed [89]. However, the use of non-viral vectors such as nanoparticles to deliver nucleic acid is a better choice as viral vectors can cause immune response and should always be treated with caution [90,91]. siRNA permits knockdown of gene expression by selectively targeting genes such as *MMP, ganglioside-monosialic acid 3 synthase (GM3S)* and *TNF-α*, which are overexpressed in chronic wounds. In vivo delivery of siRNA requires a carrier for transport into cells to protect against physiological nucleases. Nanoparticle-based technology has enabled targeted transport of siRNA and prevention from degradation [92,93]. Clinical studies delivering siRNA to cure several diseases have been promising, yet primary clinical trials were unsuccessful due to inadequate efficacy or significant off-target effects. RNAi technology demands additional refinement prior to widespread clinical use. Barriers for successful siRNA delivery for efficient therapy are degradation of siRNAs by enzymes in the wound environment and siRNAs not readily taken up by the cells due to electrostatic constraints, as the negatively charged cell wall will not easily allow penetration of negatively charged siRNAs into the cells. To address these problems, a wide variety of delivery systems have been pre-clinically tested using nanoparticles. Delivery vehicles for siRNAs such as those mentioned below have attained varying degrees of efficacy, with topical dosing and intravenous formulations, and are currently at the forefront of testing for clinical use [94,95].

(a) Ganglioside GM3 siRNA: Ganglioside GM3 is a monosialodihexosylganglioside produced by the enzyme GM3 synthase (GM3S). GM3S is key intermediary of insulin resistance which has proven to be highly expressed in human diabetic foot skin, diabetes stimulated obese mouse, hyperglycemic mouse, and mouse keratinocytes exposed to high glucose [96,97]. Randeria et al. showed that knockdown of GM3S expression in diabetic mice by AuNPs conjugated with GMS3 siRNA-based spherical nucleic acids (SNAs) reverse impaired wound healing in diabetic mice with no obvious toxicity [98].
(b) TNF-α siRNA: TNF-α is an inflammatory cytokine and it is required in limited amounts to accelerate wound healing as TNF-α is required for fibroblast proliferation, migration and wound remodeling. However, in the case of diabetic wound, uncontrolled production of TNF-α blocks the normal process of wound healing by increasing cell apoptosis, ROS and matrix degradation [99,100]. Kasiewicz et al. fabricated lipid nanoparticles encapsulated with specific TNF-α siRNA to accelerate wound healing in diabetic mice [101]. The same investigators demonstrated that topical application of lipid nanoparticles loaded with TNF-α siRNA in the diabetic wound of mice downregulated TNF-α expression by 40–50% with closure of wound significantly faster than control wound.
(c) Keap1 (Kelch-like erythroid cell-derived protein with CNC homology-associated protein 1) siRNA: In the absence of oxidative stress, the nuclear factor erythroid 2–related factor 2 (Nrf2) binds to Keap1 (in the cytoplasm, which subsequently lead to Nrf2 degradation by ubiquitination). However, in the presence oxidative stress, keap1 is covalently modified in some region that prevents degradation of Nrf2. Following which, Nrf2 enters the nucleus by dissociating from the repressor site of Keap 1 and binds to the antioxidant response element (ARE) in the promotor region of a wide variety of genes responsible for preventing oxidative stress and protein instability, as well as proteasome integrity [102]. ARE is situated in the promoter area of genetic materials that encode many antioxidant and phase II detoxifying enzymes. These enzymes are essential for cellular protection by increasing the elimination of cytotoxic electrophiles and ROS [103]. Chronic hyperglycemia in diabetes causes imbalance of ROS and over production of Keap1, leading to degradation of Nrf2, which regulates diabetic oxidative stress [104,105]. Rabbani et al. has developed a liposome and protein hybrid nanoparticulate delivery system loaded with siRNA specific to Keap1, which can accelerate diabetic wound with severe oxidative stress [94].
(d) miR-146a: The hyperglycaemic state also activates redox-sensitive transcription factors, mainly NFkB, which leads to over production of pro-inflammatory cytokines such as IL-6 and IL-8 that delay wound healing by extending the inflammation period [106,107]. Cerium oxide nanoparticles (CNP) can act as a therapeutic agent for oxidative stress as CNP has an ability to scavenge free radicals [108,109]. The initial inflammatory response to injury is essential to activate normal wound healing while sustained inflammatory response impairs wound healing associated with diabetic wounds [110]. Zgheib et al. has designed microRNA (miR-146a) loaded CNPs for diabetic wound healing [111]. miR-146a has been reported to negatively regulate the production of pro-inflammatory cytokines, implying that miR-146a can act as a molecular brake in the inflammatory response [112,113]. miR-146a suppresses interlukin-1 receptor associated kinase 1 (IRAK1) and tumor necrosis factor receptor associated kinase 6 (TRAF6), which induces overexpression of IL-6 and IL-8 [114,115]. Down regulation of miR-146a, which influences the upregulation of its target gene IRAK1 and TRAF6, has been observed in diabetic wounds [116]. CNP-miR-146a has been reported to be effective for diabetic wound healings [111]. The use of nanoparticles as a delivery system for siRNA (as summarized in Table 2) may be able to overcome the boundaries of existing methods of free siRNA delivery at wound site because of the capability for encapsulation, controlled release, specific targeting, stability, and bioavailability.

### 5.3. Antibiotics

The most common characteristic of prolonged chronic wound healing is infection. In diabetic wounds, surface infections lead to the development of biofilms superficially within the wound, disrupting normal physiological wound healing [117]. Contamination by pathogens in a wound can evolve into colonization of bacteria, leading to localized infection and even systemic infection, sepsis and multi-organ dysfunction [118]. The presence of a biofilm leads to prolonged inflammation by stimulation of NO, cytokines and free radicals [119]. Hence, an effective treatment is required to deliver antimicrobial drugs to infected wounds for normal wound healing. In this regard, nanoparticles can be utilized to specifically target and eliminate pathogens. The antimicrobial effect of nanoparticles comprises destruction of cell membranes, impediment of enzyme pathways, modifications of microbial cell wall and nucleic materials pathway, and as a delivery system.

AgNPs have demonstrated a huge potential for different biomedical applications, such as in detection and diagnosis, drug delivery, coating of biomaterials, devices for novel antimicrobial agents and in regeneration materials [59]. For instance, AgNPs are known to have antimicrobial activity, which when incorporated with EGF, promotes re-epithelization, resulting in wound healing in diabetic mice [120]. AgNPs embedded in cellulose nanocrystals (CNCs) isolated from *syzygium cumini* leaves (which help to preserve the moist environment in the wound) has accelerated wound healing in diabetic mice [121].

Nanoparticle encapsulation with antimicrobial drug has developed as a novel and capable alternative to address diabetic wound infection with minimal undesirable side effects [122]. A major challenge faced in antibiotic therapy is antibiotic resistance. According to the World Health Organization (WHO), Methicillin-resistant *Staphylococcus aureus* (MRSA) infections have caused a higher mortality in patients by 64% compared to the non-resistant form [123]. To overcome the challenges of multi-drug resistant bacteria and to restore the efficacy of antibiotics, Kalita et al. designed lysozyme capped gold nanoclusters (AUNC-L) functionalized with a widely used β-lactam antibiotic, ampicillin, as a model drug to combat MRSA resistance against ampicillin and to accelerate diabetic wound with MRSA persistent infection [117]. Free ampicillin has failed to reduce MRSA infection on diabetic wounds while AUNC-L-Amp has accelerated wound healing by eliminating the MRSA persisted infection within the wound [117]. This same study showed that metallic nanoclusters in combination with antibiotics, augment their antibacterial properties and thereby mitigate the cytotoxicity of both the agents by reducing the necessity for high drug dosages. For the development of nano-antibiotics against microbial pathogens, toxicity of non-natural materials is a limiting step for utilization in clinical application.

**Figure 6:**
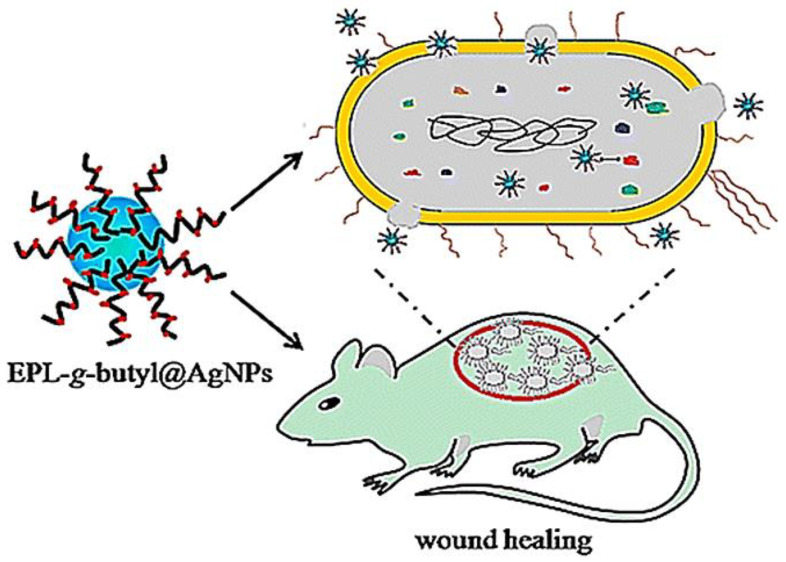
Nanocomposite (EPL-*ց*-butyl@AgNPs) shows effective antibacterial activity against both Gram-negative (*P. aeruginosa*) and Gram-positive (*S. aureus*) bacteria without the emergence of bacterial resistance, which effectively promoted infected wound healing in diabetic rats. Reproduced from [124], with permission from American Chemical Society, 2016.

The emergence of bacterial resistance to conventional antibiotics represents a general challenge in clinical trials. Dai et al. developed an AgNPs-coated *ε*-Polylysine (EPL-*ց*-butyl@AgNPs) bacterial binding nanocomposite, in which *ε*-Polylysine was used to coat AgNPs so as to act as bacterial affinity ligand to combat multiple-drug resistance bacteria. The nanocomposites and levofloxacin were introduced in the culture of Gram-negative (*P. aeruginosa*) and Gram-positive (*S. aureus*) bacteria, respectively. After 30 passages, MIC remained the same for EPL-*ց*-butyl@AgNPs, while the MIC value of levofloxacin increased from 0.64 to 78 μg mL^−1^ against *S. aureus* and from 3.2 to 156 μg mL^−1^ against *P. aeruginosa*. Compared with the antibiotic, no antimicrobial resistance was detected against the EPL-*ց*-butyl@AgNPs nanocomposite, providing a promising solution to control and prevent drug resistance. Furthermore, the same investigators proved that EPL-*ց*-butyl@AgNPs offer effective antibacterial effect and wound-healing acceleration in diabetic rats by the synergetic effect of *ε*-Polylysine and AgNPs [124] (Figure 6).

According to the American Diabetes Association, 25% of hyperglycaemic patients experience delayed wound healing. Chronic wound infections are frequently polymicrobial, whereby several microorganisms share a common niche [125]. Polymicrobial wound infections usually necessitate increased doses of antibiotics and fungicides. Yet, continued antimicrobial treatments are related with possible systemic side effects and possible risk of developing drug-resistant microorganisms. Hence, Thattaruparambil-Raveendran et al. has developed chitosan (CH) bandages using fibrin nanoparticles (FNPs) encapsulated with antimicrobial agents, such as ciprofloxacin and fluconazole (cFNPs+fFNPs−CH) and demonstrated significant reduction in microbial contamination with accelerated wound healing, as compared to control animals with topical application of cFNPs+fFNPs−CH in vivo. Also, this same study analyzed the antimicrobial ability of the bandages containing nanoparticles-loaded antibiotics against a co-culture of *S. aureus, E. coli,* and *C. albicans*, to mimic the clinical scenario of polymicrobial infection in chronic wounds. The findings verified that the chitosan bandages had significant antimicrobial property towards co-cultures of bacteria and fungi, indicating that this bandage is a potential candidate for clinical applications for diabetic wound healing [126]. Liang et al. established a glycidyl methacrylate functionalized quaternized chitosan (QCSG) and gelatin methacrylate (GM) hydrogel, encapsulated with graphene oxide (GO), for drug-resistant bacterial infective wound healing. Development of injectable conductive nanocomposite hydrogel dressings based on GO and cationic polymer for wound healing is highly promising as the QCSG/GM/GO hydrogels demonstrated 95% killing ratio against *S. aureus* and *E. coli*, and for clinical drug-resistant bacterium MRSA, the bacterial killing ratio is also higher than 90%. Based on the known photothermal effectiveness of these hydrogels, near-infrared light-assisted photothermal antimicrobial activity was analyzed. Infrared irradiation of QCSG/GM/GO hydrogel for more than 10 min had killing ratios of almost 100% for all three bacteria, affirming the effective near-infrared-assisted photothermal antibacterial properties of QCSG/GM/GO hydrogels. In order to evaluate the continuous drug release ability of hydrogels, an inhibition zone assay was conducted to assess the antimicrobial activity of the doxycycline that was released from hydrogels. Inhibition for *S. aureus* and MRSA lasted for 9 days, which further confirmed sustained drug release of the hydrogels. Cell compatibility data demonstrated higher L929 cell viability with increase in incubation time for the hydrogel groups. It was noted that IL-6 expression (a biological cytokine plays a significant role in inflammatory response and secreted by several types of cells) in the wounds of the hydrogel-treated group was lower than that of the Tegaderm group on the third day, while inflammation was significantly reduced on the 7th day. Moreover, injectable QCSG/GM/GO hydrogels with antibiotics accelerated infectious skin defect wounds compared to commercially available Tegaderm with an increase in collagen deposition and re-epithelialization [127].

Bacterial infection and prolonged inflammation is a very important factor in preventing successful clinical intervention for diabetic wound healing. The above discussed research studies evaluated the antibacterial property of nanoparticles loaded with antibiotics (summarized in Table 3). The findings that showed the effective antibacterial property of nanoparticulate systems against major drug-resistant bacteria may give rise to novel clinical applications in the near future.

### 5.4. Antioxidants

In the inflammatory phase of wound healing, neutrophils, leucocytes, and monocytes will be attracted to the wound sites by biologically active mediators and then attack the microorganisms and foreign debris via phagocytosis, which will lead to the production of ROS [128]. The antioxidant system in the cell evolves to play central roles in scavenging these free radicals to maintain redox homeostasis or the equilibrium between free radicals and antioxidants [129]. ROS including superoxide (O_2_-), hydrogen peroxide (H_2_O_2)_, hydroxyl radical, and other reactive oxygen derivatives, are very lethal and cause extensive damage to protein, DNA and lipids, thereby affecting normal cellular functioning [130]. ROS is produced in the cell as an unavoidable by-product of oxidative phosphorylation [131]. ROS is constantly being generated at basal levels. However, they are unable to cause damage, as they are being scavenged by different antioxidant mechanisms [132]. As high levels of ROS can damage cells by oxidizing lipids and proteins, the levels are tightly controlled by the presence of ROS scavenging enzymes and small molecule antioxidants [133]. Altered redox signaling (non-equilibrium between free radicals and antioxidants) that leads to oxidative stress is widely accepted as a contributor to the development of diabetic complications, including cardiovascular disease, nephropathy and retinopathy [134,135]. Accumulation of ROS leads to significant destruction of endogenous stem cells, growth factors, and nucleic acids in the wounded tissue, thus greatly affecting their regenerative potential, causing delayed wound healing [136].

Nanoparticles-based treatment has shown promising results in promoting antioxidant activities in diabetic rodents for effective wound healing. Bairagi et al. has developed PLGA nanoparticles encapsulated with ferulic acid (FA; 4-hydroxy-3-methoxycinnamic acid) to study its effect in diabetic wound healing. FA is a phenolic compound and a natural antioxidant with a potential synergistic therapeutic effect in diabetic wound healing due to its hypoglycemic, free radical scavenging, angiogenic, antibacterial, and neurogenic effects. In this same study, the investigators demonstrated that FA-loaded polymeric nanoparticles dispersion (oral administration) and FA-loaded polymeric nanoparticles-based hydrogel (topical administration) treated wounds had faster epithelization of the wound, leading to effective wound closure on day 14 as compared with the diabetic wound group [137]. The formation of advanced glycation end products (AGEs) has been recognized as an important pathophysiological mechanism in the development of diabetic ulcers; the binding of circulatory AGE to RAGE (receptor for AGEs) on different cell types leads to impaired function of growth factors. Glycation is an important pathway in the pathogenesis of microvascular and macrovascular complications of DFUs. AGE and RAGE result in oxidative stress and cause abnormal angiogenesis in wound healing [138]. In type 2 diabetic skin tissues, the expression of both AGE and RAGE were increased when compared with normal skin tissues. Moreover, a study on human dermal fibroblasts demonstrated that cell arrest and apoptosis was increased [139]. The levels of nitric oxide were increased in glycated soluble protein (AGE-BSA) treated kidney cell lines, suggesting oxidative stress [140]. The blockage of RAGE by intraperitoneal soluble RAGE, significantly suppressed the TNF-α and IL-6 while enhancing cutaneous wound closure in db/db mice [141]. A previous study reported that an antioxidant, epigallocatechin gallate (EGCG) decreased RAGE mRNA and protein expression in AGE-treated human mesangial cells [142]. EGCG also attenuated AGE-induced RAGE in neuronal cells [143,144], and alpha-lipoic acid (ALA) is a scavenger of many ROS [144]. Chen et al. demonstrated that the combination of antioxidants EGCG, ALA and AuNPs in specific concentrations significantly decreased expression of the RAGE protein within cultured fibroblasts (Hs68) and diabetic wound healing in a mouse model. In this study, the authors showed that a mixture of AuNP, EGCG and ALA (AuEA) significantly decreased AGE-induced RAGE protein expression in fibroblasts (Hs68). Furthermore, topical AuEA application decreased RAGE expression in diabetic mouse skin, which suggests that a combination of EGCG, ALA and AuNPs considerably accelerated diabetic wound healing through anti-inflammatory and angiogenesis via modulation of antioxidants [145]. Similarly, topical gas-injection of a EGCG and AuNP liquid mixture (AuE) using the GNT GoldMed™ liquid DDS showed a significantly higher rate of wound closure on wild-type and streptozotocin-induced diabetic mouse skin, associated with increased epidermal growth factor receptors and VEGF, which stimulate wound recovery and the new tissue formation. Besides, collagen I, III and hyaluronic acid protein expressions increased in the wound area. These are essential factors of physiological matrix and wound healing [146]. In another study, Ponnanikajamideen et al., using the leaf extract powder of a plant, *Chamaecostus Cuspidatus*, and fabricated green synthesized AuNPs, showed 50% inhibition of free radicals by green synthesized AuNPs without inducing any lethal effects in a mouse model, with restoration of blood glucose, glycogen and insulin levels in the diabetic mice after 21 days of treatment [147]. He et al. fabricated PCL and quaternized chitosan-*ց*-polyaniline (QCSP) nanofibers to promote wound healing [128]. The nanofibrous wound dressings displayed comparable mechanical characteristics to soft tissue, free radical scavenging capability, antimicrobial property and biocompatibility. Their data suggested that the antioxidant capability of PCL/QCSP15 nanofibers heightened with increasing concentration of QCSP and almost 70% of free radicals can be cleared by 6 mg mL^−1^ of PCL/QCSP15 dispersion liquid, and the scavenging efficacy for DPPH has shown more than 80% when the content of PCL/QCSP15 dispersion liquid reached 8 mg mL^−1^. Furthermore, wounds that received treatment by PCL/QCSP15 nanofiber dressing showed elevated collagen secretion, granulation tissue thickness and enhanced angiogenesis, leading to accelerated wound closure compared to commercially available Tegaderm [128]. As DFU remain a complex problem in clinical settings, the above discussed studies (highlighted in Table 4) strongly support the beneficial effects of anti-oxidants and nanoparticles on diabetic patients with cutaneous wounds and clearly provide a basis for the potential therapeutic application of AuEA, PLGA nanoparticles in chronic wound therapy.

## 6. Regulatory Pathway for Nanomaterial

Nanotechnology is an emerging technology that can be used in a broad array of FDA-regulated products. There are two main points for consideration when providing an initial screening tool that can be applied to FDA-regulated nanotechnology products. 1. Whether an engineered material is in nanoscale range of 1 to 100 nm with at least one external dimension. 2. Whether an engineered material demonstrates properties involving physical characteristics or biological effects that are attributable to its dimensions, even if these dimensions fall outside the nanoscale range, up to 1 µm (1000 nm). FDA regulatory framework and review process adequately identify and manage potential risk associated with the use of nanomaterials in products [148,149]. The safety assessment and the toxicity and biocompatibility of nanomedicines go through the same FDA regulatory process as drugs that do not contain nanotechnology products. Primary development of a nanotech product is at the nexus of basic and preclinical research, where further development often includes collaborations among academic supervisor and industrial researchers. Primary studies may be initial tests for its translational potential and will offer a base for further preclinical development, which involves tests that meet the regulatory requirements for investigational new drug (IND) applications, new drug applications (NDAs), and abbreviated new drug applications (ANDAs) by the United States FDA [150]. After gaining the status of a new research drug, to administer an investigation drug or biologic to humans from IND, nanomedicines or nano DDSs, investigations are initiated to evaluate their safety and efficacy in humans by clinical trials. These clinical trials are divided into three phases: phase 1 (mainly assesses safety), phase 2 (mainly determines efficacy) and phase 3 (safety, efficacy and dosage are evaluated). After obtaining approval in these three phases, the IND can be filed by the FDA to request endorsement of the new nanomedicine or nano DDSs [151,152]. FDA regulations, as well, specifically address nanomaterials safety, for which it is essential to explore the properties to understand the mechanisms by which nanomaterials communicate with biological systems to identify exposure, hazards and their possible risks [153]. Biocompatibility is an essential property in the design of nanomaterial-based DDSs. Biocompatibility is defined as material that has the potential to perform the desired function in a specific application and its surface would not elicit any undesired response from the organisms [154]. Pre-clinical evaluation of nanomaterials goes through a complete biocompatibility testing that includes in vivo studies followed by essential in vitro assays to prove its biocompatibility, so as to avoid toxicology concerns [155]. The pharmacokinetics and distribution of nanoparticles in the body depends on their surface physicochemical characteristics, shape and size. For example, nanoparticles that are 10 nm in size, are observed in blood, liver, spleen, kidney, testis, thymus, heart, lung, and brain, whilst larger particles are found only in the spleen, liver, and blood [156]. The surface properties of nanoparticles also affect their distribution in these organs, since combination with serum proteins available in the systemic circulation may influence cellular uptake. It should be reiterated that a biocompatible material does not elicit any physiological immune response. One of the reasons that an immune response is triggered is due to possible adsorption by body proteins, therefore, evaluation of an in vivo protein profile is essential to address the biological interactions and to establish its biocompatibility [157]. Lastly, clearance of nanoparticles is also dependent on size and surface of nanoparticles. Nanoparticles that are below 10 nm size are promptly cleared by renal excretion, whereas nanoparticles larger than 200 nm are efficiently taken up by mononuclear phagocytic system located in the liver, spleen, and bone marrow [158]. Studies are therefore required to address how nanomaterials penetrate cells and tissues, and the respective biodistribution, degradation, and excretion before translation into clinical applications.

## 7. Clinical Status of Nanomedicine

Several agents for promoting tissue healing, such as growth factors, small molecules, and siRNA-based therapeutics, have shown promising results in improving wound healing in preclinical trials. Despite recent advances, challenges in retention and duration of the therapeutic effect in the harsh wound environment, has limited the pace for clinical implementation. Nanoparticle formulations, nanofibrous scaffold and hydrogel-related treatments are being devised to overcome this limitation. Ultimately, these technologies will require additional validation by testing in larger animal models, particularly the porcine model, before the consideration of a clinical setting [35,159]. AgNPs have been used for numerous clinical trials in the therapy of wounds, especially burns and chronic wounds (diabetic wounds). Currently, there are some commercially available dressings containing AgNPs [160]. Among the different polymers developed to fabricate polymeric nanoparticles, PLGA is one of the most successfully used synthetic polymers, with FDA approval for clinical use in humans as a DDS, due to the following desirable properties: (1) well-described formulations and methods of production adapted to various types of drugs, ranging from small molecules to macromolecules; and (2) ability to protect drugs from degradation and the possibility of sustained release [161]. Recombinant human-PDGF (rhPDGF), the only FDA-approved growth factor available for clinical use, has been shown in clinical trials to increase the incidence of complete wound closure and decrease the time to achieve complete wound healing [162]. The only siRNA delivery depot in clinical pipeline is the siG12D LODER therapeutic to combat non-resectable pancreatic dual adenocarcinoma [163]. In the market, modern wound bandage materials that are effective for skin regeneration have arrived. Despite the demand for the use of improved dressing materials for wound healing, many of the wound healing material that are applied clinically rely on safety data and experience rather than the efficacy rate. Inorganic-based Au, copper, ZnO, cerium oxide, and silica nanoparticles are still under clinical investigation [164].

## 8. Future Perspective

The usage of nanoparticle-based treatments by incorporation of therapeutic drugs and siRNAs, is an exciting and novel field for wound treatment, with unlimited prospects and opportunities. Nanoparticle-based remedies involve delivery of therapeutic drugs that promote wound healing, due to the integral properties of the nanoparticles as efficient delivery systems. There are promises of achieving greater efficacy and specificity, with a smaller amount of systemic side effects. In addition, compared to conventional antibiotics, nanoparticle-based antimicrobial treatment is more likely to eradicate bacteria developing resistance. However, the adverse biological effects elicited by nanoparticles should be further investigated and the development of nanoparticle-based therapies should be undertaken with a reasonable amount of caution, bearing in mind nanosafety concerns. Working towards improving the efficacy of nanoparticle wound treatments should go hand in hand with investigating the long and short-term effects of nanoparticle-based treatments, as well as the mechanisms underpinning them. The current approach of exploiting nanotechnology for the treatment of diabetic wound healing is occurring at an exponential rate. Further research and development efforts in this emerging field will have a positive impact on the treatment of wound regeneration, especially chronic wounds, which pose a significant burden on the quality of life and healthcare. Therefore, it is likely that nanotechnology-based remedies will possibly be the next frontier poised for breakthroughs in meeting the clinical needs of chronic wound healing.

## 9. Conclusions

Wound healing is an intricate three-staged process involving inflammation, proliferation and remodeling. The physiology of the healing process is perturbed in the case of DFU by both internal and external factors, such as altered cellular and cytokines response, poor vascularization, and infection by microorganisms. This overview focusing on nanoparticle-based therapeutics that deliver peptides; nucleic acids; antibiotics; and antioxidants incorporated in polymeric and natural nanostructures, hydrogels and nanofibers, have revealed promising results on re-epithelialization, deposition of collagen fibers, tissue regeneration, and ultimately a faster rate of wound closure in chronic diabetic wounds. Moreover, studies have clearly shown the effective antibacterial property of nanoparticulate systems against major drug-resistant bacteria. The combination of nanoparticles and biopolymers as a nanocomposite have a greater effect in speeding up tissue repair and wound healing. The use of nanomaterials for wound healing has been widely explored, although it is still far from commercialization and routine clinical practice. However, the studies collated in this review may provide more insight for pre-clinical testing of nanoparticle-based therapeutics for DFU, before instituting the relevant clinical trials and further commercialization. The overall outlook of nanoparticle DDSs is promising, as they are being developed not only for treatment of diabetic wounds, but many other diseases including cancer.

## Figures and Tables

**Table 1:** Nanoparticles-based therapeutic incorporated with growth factors for diabetic wound healing.

Type of Nanoparticles	Incorporated Growth Factors	In-Vitro Model	In-Vivo Model	Results	Route of Administration	Ref.
SLN and NLC nanoparticles	rhEGF	Fibroblasts, keratinocytes	8 mm in diameter skin wound was created in diabetic male db/db mice	rhEGF loaded lipid nanoparticles exhibited higher fibroblast and keratinocyte proliferation and greater resolution of inflammation re-epithelialization and significant wound closure compared to free rhEGF	Topical SLN-rhEGF and NLC-rhEGF dressing of nanoparticles at wound site	[81]
PLGA nanoparticles	VEGF, bFGF	_	8 mm in diameter skin wound was created in diabetic male db/db mice	VEGF and bFGF loaded nanoparticles treated wound stimulated significant granulation tissue formation, collagen secretion and re-epithelialization, and accelerated wound closure compared to controls and NPs without biomolecules	Topical dressing of nanoparticles incorporated polydimethylsiloxane/fibrin-based scaffold at wound site	[82]
NaCMCh nanoparticles	rhEGF	Fibroblast	20 mm in diameter skin wound was created in diabetic male Sprague–Dawley rats	Nanoparticles-treated cells showed higher cell viability with enhanced wound healing rate when compared to controls and free rhEGF	Topical dressing of nanoparticles incorporated chitosan-based hydrogel at wound site	[83]
Gelatin nanoparticles	VEGF, PDGF, bFGF, EGF	Human umbilical vein endothelial cells (HUVEC)	15 mm in diameter skin wound was created in diabetic male Sprague–Dawley rats	Gelatin nanoparticles loaded with multiple angiogenic growth factors showed high cell proliferation and accelerated complete healing along with enhanced collagen synthesis, re-epithelialization and vascularization compared to controls	Topical dressing of drug loaded collagen/ hyaluronic acid nanofibrous scaffold at wound site.	[84]
AuNPs	KGF	Keratinocytes	10 mm in diameter skin wound was created in diabetic rats	KGF-AuNPs increased healing effect compared to free KGF and nanoconjugate promoted re-epithelialization and wound contraction along with elevated expression of Col-I, α-SMA and TGF-β1, leading to accelerated wound healing compared to controls	Topical gelatin hydrogel dressing encapsulated with KGF-AuNPs	[85]

**Table 2:** Nanoparticles-based therapeutics encapsulated with nucleic acids for diabetic wound healing.

Type of Nanoparticles	Encapsulated Nucleic Acids	In-Vitro Analysis	In-Vivo Model	Results	Route of Administration	Ref.
Cationic lipid nanoparticles	Keap1 siRNA	Knockdown of Keap1 via LPP-10 correlated with an increased protein expression of Nrf2, a critical transcription factor in maintenance of cell integrity	10 mm in diameter skin wound was created in diabetic mice	Nanoparticles treatment complexing siKeap1, restored Nrf2 antioxidant function, accelerated diabetic tissue regeneration, and augmented reduction oxidation homeostasis in the wound environment	Topical administration to wound	[94]
AuNPs	GM3S siRNA based SNAs	GM3S loaded nanoparticles treated wounds were completely closed in hyperglycemic conditions and almost closed in normoglycemic medium	6 mm in diameter skin wound was created in diet- induced obese C57BL/6 diabetic mice	Nanoparticles treated wound stimulated granulation of tissue area, vascularization, and IGF1 and EGF receptor phosphorylation are elevated in GM3S SNA-treated wounds that accelerated active wound closure compared to free GM3S siRNA	Topical administration to wound	[98]
Lipid nanoparticles	TNF-α siRNA	-	8 mm in diameter skin wound was created in diabetic C57BL/6 mice	Nanoparticles in diabetic mice accelerated TNF-α knockdown of diabetic wound that elevated the wound closure rate within 13 days, which was statistically faster than control wounds, which remained open on Day 16	Topical administration to wound	[101]
CNPs	miR-146a	-	8 mm in diameter skin wound was created in Db/Db mice	CNP-miR-146a improves wound healing in diabetic mice wound model without compromising wound strength and elasticity	Topical administration to wound	[111]

**Table 3:** Nanoparticles-based therapeutics incorporated with an antibiotic for diabetic wound healing.

Type of Nanoparticles	Incorporated Antibiotics	Antibacterial Assay	In Vitro Analysis	In Vivo Model	Results	Route of Administration	Ref.
AUNC-L	Ampicillin	50–89-fold increase in antibacterial activity of nanoclusters compared to Free-Amp in terms of zone of inhibition against 9 non-resistant bacterial pathogens	Cyto-compatibility study of nanoclusters with human blood cells and fibroblast shows higher cell viability compared to free ampicillin	1.5 cm in diameter skin wound was created in diabetic male Wistar rats followed by MRSA infection	Nanoclusters eradicated MRSA infections from diabetic wound which accounts for pronounced and faster wound healing	Topical application of nanoclusters to wound	[117]
AgNPs coated *ε*-Polylysine nanocomposites	*ε*-polylysine	4.7 μg mL^−1^ of nanocomposite inhibited antibiotic resistant Gram-negative and Gram-positive bacteria	Fibroblast cells maintained a viability of 80% after 2 days treatment with nanoparticles	1.5 cm in diameter skin wound was created in diabetic male Wistar rats followed by inoculation with *P. aeruginosa* and *S. aureus*	Nano-composite modulated inflammation of cells primes to wound healing acceleration without side effects on dermal tissues, eliminating the infection in wound	Topical application of nanoparticles with antibiotic to wound	[124]
FNPs	Ciprofloxacin, fluconazole	The nanoparticles loaded bandages showed promising inhibitory activity against individual and co-culture *S. aureus, E. coli*, and *C. albicans*	Toxicity of the bandages studied against the human dermal fibroblast cell line proved its cyto-compatibility	1.5 cm in diameter skin excisional infected wounds model was created in diabetic female Sprague–Dawley rats by inoculation of *S. aureus*, *E. coli*, and *C. albicans*	The bandages with nanoparticles showed a significant reduction in microbial populations in the poly-microbial infected rat wound model which accelerated wound healing	Topical dressing of bandage with nanoparticles loaded antibiotic to wound	[126]

**Table 4:** Nanoparticles-based therapeutics incorporated with antioxidants for diabetic wound healing.

Type of Nanoparticles	Incorporated Antioxidants	In Vitro Analysis	In Vivo Model	Results	Route of Administration	Ref.
PLGA nanoparticles	FA	-	2.5 cm in length skin wound was created in diabetic Wistar rats of either sex	FA nanoparticles topical and oral treatment is effective in promoting wound healing in diabetic rats	Oral administration of nanoparticles and topical administration of hydrogel containing nanoparticles	[137]
AuNPs	EGCG, ALA	Antioxidant loaded nanoparticles group significantly decreased RAGE expression in AGE-treated fibroblast cells	1 cm in diameter skin wound was created in diabetic male BALB/c mice	Nanoparticles loaded with antioxidant significantly increased the rate of diabetic wound healing by decreasing RAGE expression than control and free antioxidants	Topical application of nanoparticles containing antioxidant on wound	[145]
AuNPs	EGCG	-	1 cm in diameter skin wound was created in diabetic male BALB/c mice	Epidermal growth factor receptor and collagen I and III protein expression, and hyaluronic acid expression increased considerably which significantly increases the rate of wound healing both in wild-type and diabetic mice	Nanoparticles containing liquid by gas-injection applied directly to the wound	[146]
AuNPs	Green synthesis of AuNPs by *Chamaecostus Cuspidatus*	-	Blood sampling in Wistar male diabetic mice	Nanoparticles with nontoxic effects showed 50% inhibition of free radicals with restoration of blood glucose, glycogen, and insulin levels in the diabetic mice	Intraperitoneal administration of green synthesized nanoparticle	[147]

## Abbreviations

AGEs: Advanced glycation end products; AgNPs, Silver nanoparticles; ALA, Alpha lipoic acid; α-SMA, Alpha smooth muscle actin; ANDAs, Abbreviated new drug applications; ARE, Antioxidant response element; AuE, Gold nanoparticle liquid mixture; AuEA, Gold nanoparticle + EGCG and ALA; AUNC-L, Lysozyme capped gold nanoclusters; AuNPs, Gold nanoparticles; bFGF, Basic fibroblast growth factor; *C. albicans, Candida albicans*; CNCs, Cellulose nanocrystals; CNP, Cerium oxide nanoparticles; CH, Chitosan; cFNPs+fFNPs−CH, CH bandage using fibrin nanoparticles encapsulated with ciprofloxacin and fluconazole; Col, Collagen; DDSs, Drug-delivery systems, DFU, Diabetic foot ulcer; DM, Diabetes mellitus; EGF, Endothelial growth factor; EGCG, Epigallocatechin gallate; EPL-ց-butyl@AgNPs, Silver nanoparticle coated ε-Polylysine; ECM, Extracellular matrix; *E. coli, Escherichia coli*; FA (4-hydroxy-3-methoxycinnamic acid), Ferulic acid; FNPs, Fibrin nanoparticles; FGF, Fibroblast growth factor; FDA, Food and Drug Administration; GM, Gelatin methacrylate; GM3S, Ganglioside-monosialic acid 3 synthase; GF, Growth factor; GO, Graphene oxide; HA, Hyaluronic acid; HUVEC, Human umbilical vein endothelial cells; H_2_O_2_, Hydrogen peroxide; IDF, International Diabetes Federation; IL-6, Interleukin 6; IND, Investigational new drug; IRAK1, Interlukin-1 receptor associated kinase 1; Keap1, Kelch-like erythroid cell-derived protein with CNC homology-associated protein 1; KGF, Keratinocyte growth factor; miR, Micro RNA; MMP, Matrix metalloproteinase; MRSA, Methicillin-resistant *Staphylococcus aureus*; NaCMCh, Sodium carboxymethyl chitosan; NADP, Nicotinamide adenine dinucleotide phosphate; NDAs, New drug applications; NLC, Nanostructure lipid carrier; NO, Nitric oxide; Nrf2, Nuclear factor erythroid 2–related factor 2; O_2_-, Superoxide; PAD, Peripheral arterial disease; *P. aeruginosa, Pseudomonas aeruginosa*; PDGF, Platelet derived growth factor; PCL, Poly (ε-caprolactone); PEG, Poly (ethylene glycol); PLA, Poly (lactic acid); PLGA, Poly (lactic-co-glycolic acid); QCSG, Glycidyl methacrylate functionalized quaternized chitosan, QCSP, Quaternized chitosan-ց-polyaniline; RAGE, receptor for AGEs; rhEGF, Recombinant human EGF; rhPDGF, Recombinant human-PDGF; ROS, Reactive oxygen species; *S. aureus, Staphylococcus aureus*, SLN, Solid lipid nanoparticles; SNAs, SiRNA-based spherical nucleic acids; TGF-β, Transforming growth factor beta; TIMPs, Tissue inhibitor of metalloproteinases; TNF-α, Tumor necrosis factor alpha; TRAF6, Tumor necrosis factor receptor associated kinase 6; VEGF, Vascular endothelial growth factor; WHO, World Health Organization; Zn, Zinc; ZnO, Zinc oxide.

## References

[1] Kasiewicz L.N., Whitehead K.A. (2017). Recent advances in biomaterials for the treatment of diabetic foot ulcers. Biomater. Sci..

[2] Noor S., Zubair M., Ahmad J. (2015). Diabetic foot ulcer-A review on pathophysiology, classification and microbial etiology. Diabetes Metab. Syndr..

[3] International Diabetes Federation IDF Diabetes Atlas 9th Edition 2019, Global Estimates for the Prevalence of Diabetes for 2019, 2030 and 2045. http://www.diabetesatlas.org/.

[4] Armstrong D.G., Boulton A.J.M., Bus S.A. (2017). Diabetic Foot Ulcers and Their Recurrence. New Engl. J. Med..

[5] Forlee M. What is the diabetic foot? The rising prevalence of diabetes worldwide will mean an increasing prevalence of complications such as those of the extremities. http://www.cmej.org.za/index.php/cmej/article/view/1770.

[6] Wu S.C., Driver V.R., Wrobel J.S., Armstrong D.G. (2007). Foot ulcers in the diabetic patient, prevention and treatment. Vasc. Health Risk Manag..

[7] Frykberg R.G., Zgonis T., Armstrong D.G., Driver V.R., Giurini J.M., Kravitz S.R., Landsman A.S., Lavery L.A., Moore J.C., Schuberth J.M. (2006). Diabetic foot disorders. A clinical practice guideline (2006 revision). J. Foot Ankle. Surg..

[8] Bowering C.K. (2001). Diabetic foot ulcers. Pathophysiology, assessment, and therapy. Can. Fam. Phys..

[9] Tang W.H., Martin K.A., Hwa J. (2012). Aldose reductase, oxidative stress, and diabetic mellitus. Front. Pharmacol..

[10] Clayton W., Elasy T.A. (2009). A Review of the Pathophysiology, Classification, and Treatment of Foot Ulcers in Diabetic Patients. Clin. Diabetes.

[11] Simmons Z., Feldman E.L. (2002). Update on diabetic neuropathy. Curr. Opin. Neurol..

[12] Juster-Switlyk K., Smith A.G. (2016). Updates in diabetic peripheral neuropathy. F1000Research.

[13] Avogaro A., Albiero M., Menegazzo L., de Kreutzenberg S., Fadini G.P. (2011). Endothelial dysfunction in diabetes: The role of reparatory mechanisms. Diabetes Care.

[14] Gonzalez A.C., Costa T.F., Andrade Z.A., Medrado A.R. (2016). Wound healing—A literature review. An. Bras. Dermatol..

[15] Iqbal A., Jan A., Wajid M.A., Tariq S. (2017). Management of Chronic Non-healing Wounds by Hirudotherapy. World J. Plast. Surg..

[16] Cañedo-Dorantes L., Cañedo-Ayala M. (2019). Skin Acute Wound Healing: A Comprehensive Review. Int. J. Inflamm..

[17] Nurden A.T. (2011). Platelets, inflammation and tissue regeneration. Thromb. Haemost..

[18] Ng M.F. (2010). The role of mast cells in wound healing. Int. Wound J..

[19] Theoharides T.C., Kempuraj D., Tagen M., Conti P., Kalogeromitros D. (2007). Differential release of mast cell mediators and the pathogenesis of inflammation. Immunol. Rev..

[20] Shah J.M., Omar E., Pai D.R., Sood S. (2012). Cellular events and biomarkers of wound healing. Indian J. Plast. Surg..

[21] Guo S., Dipietro L.A. (2010). Factors affecting wound healing. J. Dent. Res..

[22] Li J., Chen J., Kirsner R. (2007). Pathophysiology of acute wound healing. Clin. Dermatol..

[23] Van De Water L., Varney S., Tomasek J.J. (2013). Mechanoregulation of the Myofibroblast in Wound Contraction, Scarring, and Fibrosis: Opportunities for New Therapeutic Intervention. Adv. Wound Care.

[24] Marshall C.D., Hu M.S., Leavitt T., Barnes L.A., Lorenz H.P., Longaker M.T. (2018). Cutaneous Scarring: Basic Science, Current Treatments, and Future Directions. Adv. Wound Care.

[25] Monaco J.L., Lawrence W.T. (2003). Acute wound healing an overview. Clin. Plast. Surg..

[26] Patel S., Srivastava S., Singh M.R., Singh D. (2019). Mechanistic insight into diabetic wounds: Pathogenesis, molecular targets and treatment strategies to pace wound healing. Biomed. Pharmacother..

[27] Cho H., Blatchley M.R., Duh E.J., Gerecht S. (2019). Acellular and cellular approaches to improve diabetic wound healing. Adv. Drug Deliv. Rev..

[28] Xu F., Zhang C., Graves D.T. (2013). Abnormal cell responses and role of TNF-α in impaired diabetic wound healing. Biomed. Res. Int..

[29] Chitturi R.T., Balasubramaniam A.M., Parameswar R.A., Kesavan G., Haris K.T., Mohideen K. (2015). The role of myofibroblasts in wound healing, contraction and its clinical implications in cleft palate repair. J. Int. Oral. Health.

[30] Nguyen T., Mobashery S., Chang M. (2016). Roles of Matrix Metalloproteinases in Cutaneous Wound Healing.

[31] McCarty S.M., Percival S.L. (2013). Proteases and Delayed Wound Healing. Adv. Wound Care.

[32] Ayuk S.M., Abrahamse H., Houreld N.N. (2016). The Role of Matrix Metalloproteinases in Diabetic Wound Healing in relation to Photobiomodulation. J. Diabetes Res..

[33] Gottrup F., Apelqvist J. (2012). Present and new techniques and devices in the treatment of DFU: A critical review of evidence. Diabetes Metab. Res. Rev..

[34] Goyal R., Macri L.K., Kaplan H.M., Kohn J. (2016). Nanoparticles and nanofibers for topical drug delivery. J. Control. Release.

[35] Whittam A.J., Maan Z.N., Duscher D., Wong V.W., Barrera J.A., Januszyk M., Gurtner G.C. (2016). Challenges and Opportunities in Drug Delivery for Wound Healing. Adv. Wound Care.

[36] Pan R., Xu W., Yuan F., Chu D., Ding Y., Chen B., Jafari M., Yuan Y., Chen P. (2015). A novel peptide for efficient siRNA delivery in vitro and therapeutics in vivo. Acta Biomater..

[37] Yoo J.-W., Irvine D.J., Discher D.E., Mitragotri S. (2011). Bio-inspired, bioengineered and biomimetic drug delivery carriers. Nat. Rev. Drug Discov..

[38] Lipsky B.A., Hoey C. (2009). Topical antimicrobial therapy for treating chronic wounds. Clin. Infect. Dis..

[39] Hamdan S., Pastar I., Drakulich S., Dikici E., Tomic-Canic M., Deo S., Daunert S. (2017). Nanotechnology-Driven Therapeutic Interventions in Wound Healing: Potential Uses and Applications. ACS Cent. Sci..

[40] Baltzis D., Eleftheriadou I., Veves A. (2014). Pathogenesis and treatment of impaired wound healing in diabetes mellitus: New insights. Adv. Ther..

[41] Mordorski B., Rosen J., Friedman A. (2015). Nanotechnology as an innovative approach for accelerating wound healing in diabetes. Diabetes Manag..

[42] Jackson J.E., Kopecki Z., Cowin A.J. (2013). Nanotechnological Advances in Cutaneous Medicine. J. Nanomater..

[43] Andreu V., Mendoza G., Arruebo M., Irusta S. (2015). Smart Dressings Based on Nanostructured Fibers Containing Natural Origin Antimicrobial, Anti-Inflammatory, and Regenerative Compounds. Materials.

[44] Korrapati P.S., Karthikeyan K., Satish A., Krishnaswamy V.R., Venugopal J.R., Ramakrishna S. (2016). Recent advancements in nanotechnological strategies in selection, design and delivery of biomolecules for skin regeneration. Mater. Sci. Eng. C Mater. Biol. Appl..

[45] Zarrintaj P., Moghaddam A.S., Manouchehri S., Atoufi Z., Amiri A., Amirkhani M.A., Nilforoushzadeh M.A., Saeb M.R., Hamblin M.R., Mozafari M. (2017). Can regenerative medicine and nanotechnology combine to heal wounds? The search for the ideal wound dressing. Nanomedicine.

[46] Wang W., Lu K.J., Yu C.H., Huang Q.L., Du Y.Z. (2019). Nano-drug delivery systems in wound treatment and skin regeneration. J. Nanobiotechnol..

[47] Johnson N.R., Wang Y. (2015). Drug delivery systems for wound healing. Curr. Pharm. Biotechnol..

[48] Gelperina S., Kisich K., Iseman M.D., Heifets L. (2005). The potential advantages of nanoparticle drug delivery systems in chemotherapy of tuberculosis. Am. J. Respir. Crit. Care Med..

[49] Berthet M., Gauthier Y., Lacroix C., Verrier B., Monge C. (2017). Nanoparticle-Based Dressing: The Future of Wound Treatment?. Trends Biotechnol..

[50] Goh E.T., Kirby G., Jayakumar R., Liang X.J., Tan A., Hamblin M.R., Avci P., Prow T.W. (2016). Accelerated Wound Healing Using Nanoparticles. Nanoscience in Dermatology.

[51] Ezhilarasu H., Ramalingam R., Dhand C., Lakshminarayanan R., Sadiq A., Gandhimathi C., Ramakrishna S., Bay B.H., Venugopal J.R., Srinivasan D.K. (2019). Biocompatible Aloe vera and Tetracycline Hydrochloride Loaded Hybrid Nanofibrous Scaffolds for Skin Tissue Engineering. Int. J. Mol. Sci..

[52] Ramalingam R., Dhand C., Leung C.M., Ezhilarasu H., Prasannan P., Ong S.T., Subramanian S., Kamruddin M., Lakshminarayanan R., Ramakrishna S. (2019). Poly-ε-Caprolactone/Gelatin Hybrid Electrospun Composite Nanofibrous Mats Containing Ultrasound Assisted Herbal Extract: Antimicrobial and Cell Proliferation Study. Nanomaterials.

[53] Shan X., Liu C., Li F., Ouyang C., Gao Q., Zheng K. (2015). Nanoparticles vs. nanofibers: A comparison of two drug delivery systems on assessing drug release performance in vitro. Des. Monomers Polym..

[54] Ezhilarasu H., Sadiq A., Ratheesh G., Sridhar S., Ramakrishna S., Ab Rahim M.H., Yusoff M.M., Jose R., Reddy V.J. (2019). Functionalized core/shell nanofibers for the differentiation of mesenchymal stem cells for vascular tissue engineering. Nanomedicine.

[55] Gao W., Vecchio D., Li J., Zhu J., Zhang Q., Fu V., Thamphiwatana S., Lu D., Zhang L. (2014). Hydrogel containing nanoparticle-stabilized liposomes for topical antimicrobial delivery. ACS Nano.

[56] Slaughter B.V., Khurshid S.S., Fisher O.Z., Khademhosseini A., Peppas N.A. (2009). Hydrogels in regenerative medicine. Adv. Mater..

[57] Chai Q., Jiao Y., Yu X. (2017). Hydrogels for Biomedical Applications: Their Characteristics and the Mechanisms behind Them. Gels.

[58] Mauricio M.D., Guerra-Ojeda S., Marchio P., Valles S.L., Aldasoro M., Escribano-Lopez I., Herance J.R., Rocha M., Vila J.M., Victor V.M. (2018). Nanoparticles in Medicine: A Focus on Vascular Oxidative Stress. Oxid. Med. Cell Longev..

[59] Paladini F., Pollini M. (2019). Antimicrobial Silver Nanoparticles for Wound Healing Application: Progress and Future Trends. Materials.

[60] Chaloupka K., Malam Y., Seifalian A.M. (2010). Nanosilver as a new generation of nanoproduct in biomedical applications. Trends Biotechnol..

[61] Alarcon E.C., Griffith M., Udekwu K.I. (2015). Silver Nanoparticle Applications.

[62] Akturk O., Kismet K., Yasti A.C., Kuru S., Duymus M.E., Kaya F., Caydere M., Hucumenoglu S., Keskin D. (2016). Collagen/gold nanoparticle nanocomposites: A potential skin wound healing biomaterial. J. Biomater. Appl..

[63] Ding Y., Jiang Z., Saha K., Kim C.S., Kim S.T., Landis R.F., Rotello V.M. (2014). Gold nanoparticles for nucleic acid delivery. Mol. Ther..

[64] El-Gharbawy R.M., Emara A.M., Abu-Risha S.E. (2016). Zinc oxide nanoparticles and a standard antidiabetic drug restore the function and structure of beta cells in Type-2 diabetes. Biomed Pharm..

[65] Huang X., Zheng X., Xu Z., Yi C. (2017). ZnO-based nanocarriers for drug delivery application: From passive to smart strategies. Int. J. Pharm..

[66] Jin S.E., Jin H.E. (2019). Synthesis, Characterization, and Three-Dimensional Structure Generation of Zinc Oxide-Based Nanomedicine for Biomedical Applications. Pharmaceutics.

[67] Yang L., Sheldon B., Webster T. (2010). Nanophase ceramics for improved drug delivery: Current opportunities and challenges. Am. Ceram. Soc. Bull..

[68] Kraft J.C., Freeling J.P., Wang Z., Ho R.J. (2014). Emerging research and clinical development trends of liposome and lipid nanoparticle drug delivery systems. J. Pharm. Sci..

[69] Lin Y.H., Lin J.H., Hong Y.S. (2017). Development of chitosan/poly-γ-glutamic acid/pluronic/curcumin nanoparticles in chitosan dressings for wound regeneration. J. Biomed. Mater. Res. B Appl. Biomater..

[70] Blažević F., Milekić T., Romić M.D., Juretić M., Pepić I., Filipović-Grčić J., Lovrić J., Hafner A. (2016). Nanoparticle-mediated interplay of chitosan and melatonin for improved wound epithelialisation. Carbohydr. Polym..

[71] Chereddy K.K., Vandermeulen G., Préat V. (2016). PLGA based drug delivery systems: Promising carriers for wound healing activity. Wound Repair Regen..

[72] Sharma S., Parmar A., Kori S., Sandhir R. (2016). PLGA-based nanoparticles: A new paradigm in biomedical applications. TrAC Trends Anal. Chem..

[73] Stone W.L., Varacallo M. (2020). Physiology, Growth Factor. StatPearls.

[74] Barrientos S., Stojadinovic O., Golinko M.S., Brem H., Tomic-Canic M. (2008). PERSPECTIVE ARTICLE: Growth factors and cytokines in wound healing. Wound Repair Regen..

[75] Ulubayram K., Cakar A.N., Korkusuz P., Ertan C., Hasirci N. (2001). EGF containing gelatin-based wound dressings. Biomaterials.

[76] Mast B.A., Schultz G.S. (1996). Interactions of cytokines, growth factors, and proteases in acute and chronic wounds. Wound Repair Regen..

[77] Mark Saltzman W., Baldwin S.P. (1998). Materials for protein delivery in tissue engineering. Adv. Drug Deliv. Rev..

[78] Park J.W., Hwang S.R., Yoon I.S. (2017). Advanced Growth Factor Delivery Systems in Wound Management and Skin Regeneration. Molecules.

[79] Chu Y., Yu D., Wang P., Xu J., Li D., Ding M. (2010). Nanotechnology promotes the full-thickness diabetic wound healing effect of recombinant human epidermal growth factor in diabetic rats. Wound Repair Regen..

[80] Chereddy K.K., Lopes A., Koussoroplis S., Payen V., Moia C., Zhu H., Sonveaux P., Carmeliet P., des Rieux A., Vandermeulen G. (2015). Combined effects of PLGA and vascular endothelial growth factor promote the healing of non-diabetic and diabetic wounds. Nanomedicine.

[81] Gainza G., Pastor M., Aguirre J.J., Villullas S., Pedraz J.L., Hernandez R.M., Igartua M. (2014). A novel strategy for the treatment of chronic wounds based on the topical administration of rhEGF-loaded lipid nanoparticles: In vitro bioactivity and in vivo effectiveness in healing-impaired db/db mice. J. Control. Release.

[82] Losi P., Briganti E., Errico C., Lisella A., Sanguinetti E., Chiellini F., Soldani G. (2013). Fibrin-based scaffold incorporating VEGF- and bFGF-loaded nanoparticles stimulates wound healing in diabetic mice. Acta Biomater..

[83] Hajimiri M., Shahverdi S., Esfandiari M.A., Larijani B., Atyabi F., Rajabiani A., Dehpour A.R., Amini M., Dinarvand R. (2016). Preparation of hydrogel embedded polymer-growth factor conjugated nanoparticles as a diabetic wound dressing. Drug Dev. Ind. Pharm..

[84] Lai H.J., Kuan C.H., Wu H.C., Tsai J.C., Chen T.M., Hsieh D.J., Wang T.W. (2014). Tailored design of electrospun composite nanofibers with staged release of multiple angiogenic growth factors for chronic wound healing. Acta Biomater..

[85] Li S., Tang Q., Xu H., Huang Q., Wen Z., Liu Y., Peng C. (2019). Improved stability of KGF by conjugation with gold nanoparticles for diabetic wound therapy. Nanomedicine.

[86] Uchi H., Igarashi A., Urabe K., Koga T., Nakayama J., Kawamori R., Tamaki K., Hirakata H., Ohura T., Furue M. (2009). Clinical efficacy of basic fibroblast growth factor (bFGF) for diabetic ulcer. Eur. J. Dermatol..

[87] Hanft J.R., Pollak R.A., Barbul A., van Gils C., Kwon P.S., Gray S.M., Lynch C.J., Semba C.P., Breen T.J. (2008). Phase I trial on the safety of topical rhVEGF on chronic neuropathic diabetic foot ulcers. J. Wound Care.

[88] Wang C., Ma L., Gao C. (2014). Design of gene-activated matrix for the repair of skin and cartilage. Polym. J..

[89] Kwon M.J., An S., Choi S., Nam K., Jung H.S., Yoon C.S., Ko J.H., Jun H.J., Kim T.K., Jung S.J. (2012). Effective healing of diabetic skin wounds by using nonviral gene therapy based on minicircle vascular endothelial growth factor DNA and a cationic dendrimer. J. Gene Med..

[90] Dizaj S.M., Jafari S., Khosroushahi A.Y. (2014). A sight on the current nanoparticle-based gene delivery vectors. Nanoscale Res. Lett..

[91] Nayerossadat N., Maedeh T., Ali P.A. (2012). Viral and nonviral delivery systems for gene delivery. Adv. Biomed. Res..

[92] Kasiewicz L.N., Whitehead K.A. (2016). Silencing TNFα with lipidoid nanoparticles downregulates both TNFα and MCP-1 in an in vitro co-culture model of diabetic foot ulcers. Acta Biomater..

[93] Jozic I., Daunert S., Tomic-Canic M., Pastar I. (2015). Nanoparticles for Fidgety Cell Movement and Enhanced Wound Healing. J. Invest. Dermatol..

[94] Rabbani P.S., Zhou A., Borab Z.M., Frezzo J.A., Srivastava N., More H.T., Rifkin W.J., David J.A., Berens S.J., Chen R. (2017). Novel lipoproteoplex delivers Keap1 siRNA based gene therapy to accelerate diabetic wound healing. Biomaterials.

[95] Zhou J., Shum K.T., Burnett J.C., Rossi J.J. (2013). Nanoparticle-Based Delivery of RNAi Therapeutics: Progress and Challenges. Pharmaceuticals.

[96] Wang X.Q., Lee S., Wilson H., Seeger M., Iordanov H., Gatla N., Whittington A., Bach D., Lu J.Y., Paller A.S. (2014). Ganglioside GM3 depletion reverses impaired wound healing in diabetic mice by activating IGF-1 and insulin receptors. J. Invest. Dermatol..

[97] Tagami S., Inokuchi Ji J., Kabayama K., Yoshimura H., Kitamura F., Uemura S., Ogawa C., Ishii A., Saito M., Ohtsuka Y. (2002). Ganglioside GM3 participates in the pathological conditions of insulin resistance. J. Biol. Chem..

[98] Randeria P.S., Seeger M.A., Wang X.Q., Wilson H., Shipp D., Mirkin C.A., Paller A.S. (2015). siRNA-based spherical nucleic acids reverse impaired wound healing in diabetic mice by ganglioside GM3 synthase knockdown. Proc. Natl. Acad. Sci. USA.

[99] Liu R., Bal H.S., Desta T., Behl Y., Graves D.T. (2006). Tumor necrosis factor-alpha mediates diabetes-enhanced apoptosis of matrix-producing cells and impairs diabetic healing. Am. J. Pathol..

[100] Frank J., Born K., Barker J.H., Marzi I. (2003). In Vivo Effect of Tumor Necrosis Factor Alpha on Wound Angiogenesis and Epithelialization. Eur. J. Trauma.

[101] Kasiewicz L.N., Whitehead K.A. (2019). Lipid nanoparticles silence tumor necrosis factor α to improve wound healing in diabetic mice. Bioeng. Transl. Med..

[102] Ambrozova N., Ulrichova J., Galandakova A. (2017). Models for the study of skin wound healing. The role of Nrf2 and NF-κB. Biomed. Pap. Med. Fac. Univ. Palacky Olomouc. Czech. Repub..

[103] Lee J.M., Johnson J.A. (2004). An important role of Nrf2-ARE pathway in the cellular defense mechanism. J. Biochem. Mol. Biol..

[104] auf dem Keller U., Kümin A., Braun S., Werner S. (2006). Reactive oxygen species and their detoxification in healing skin wounds. J. Investig. Dermatol. Symp. Proc..

[105] Braun S., Hanselmann C., Gassmann M.G., auf dem Keller U., Born-Berclaz C., Chan K., Kan Y.W., Werner S. (2002). Nrf2 transcription factor, a novel target of keratinocyte growth factor action which regulates gene expression and inflammation in the healing skin wound. Mol. Cell Biol..

[106] Zgheib C., Hodges M.M., Hu J., Liechty K.W., Xu J. (2017). Long non-coding RNA Lethe regulates hyperglycemia-induced reactive oxygen species production in macrophages. PLoS ONE.

[107] Matough F.A., Budin S.B., Hamid Z.A., Alwahaibi N., Mohamed J. (2012). The role of oxidative stress and antioxidants in diabetic complications. Sultan Qaboos. Univ. Med. J..

[108] Chigurupati S., Mughal M.R., Okun E., Das S., Kumar A., McCaffery M., Seal S., Mattson M.P. (2013). Effects of cerium oxide nanoparticles on the growth of keratinocytes, fibroblasts and vascular endothelial cells in cutaneous wound healing. Biomaterials.

[109] Das S., Dowding J.M., Klump K.E., McGinnis J.F., Self W., Seal S. (2013). Cerium oxide nanoparticles: Applications and prospects in nanomedicine. Nanomedicine.

[110] Garash R., Bajpai A., Marcinkiewicz B.M., Spiller K.L. (2016). Drug delivery strategies to control macrophages for tissue repair and regeneration. Exp. Biol. Med..

[111] Zgheib C., Hilton S.A., Dewberry L.C., Hodges M.M., Ghatak S., Xu J., Singh S., Roy S., Sen C.K., Seal S. (2019). Use of Cerium Oxide Nanoparticles Conjugated with MicroRNA-146a to Correct the Diabetic Wound Healing Impairment. J. Am. Coll. Surg..

[112] Li S., Tan H.Y., Wang N., Zhang Z.J., Lao L., Wong C.W., Feng Y. (2015). The Role of Oxidative Stress and Antioxidants in Liver Diseases. Int. J. Mol. Sci..

[113] Mulholland E.J., Dunne N., McCarthy H.O. (2017). MicroRNA as Therapeutic Targets for Chronic Wound Healing. Mol. Ther. Nucleic Acids.

[114] Feng Y., Chen L., Luo Q., Wu M., Chen Y., Shi X. (2018). Involvement of microRNA-146a in diabetic peripheral neuropathy through the regulation of inflammation. Drug Des. Devel. Ther..

[115] Lo W.Y., Peng C.T., Wang H.J. (2017). MicroRNA-146a-5p Mediates High Glucose-Induced Endothelial Inflammation via Targeting Interleukin-1 Receptor-Associated Kinase 1 Expression. Front. Physiol..

[116] Xu J., Wu W., Zhang L., Dorset-Martin W., Morris M.W., Mitchell M.E., Liechty K.W. (2012). The role of microRNA-146a in the pathogenesis of the diabetic wound-healing impairment: Correction with mesenchymal stem cell treatment. Diabetes.

[117] Kalita S., Kandimalla R., Bhowal A.C., Kotoky J., Kundu S. (2018). Functionalization of β-lactam antibiotic on lysozyme capped gold nanoclusters retrogress MRSA and its persisters following awakening. Sci. Rep..

[118] Leaper D., Assadian O., Edmiston C.E. (2015). Approach to chronic wound infections. Br. J. Dermatol..

[119] Järbrink K., Ni G., Sönnergren H., Schmidtchen A., Pang C., Bajpai R., Car J. (2016). Prevalence and incidence of chronic wounds and related complications: A protocol for a systematic review. Syst. Rev..

[120] Choi H.J., Thambi T., Yang Y.H., Bang S., Kim B.S., Pyun D.G., Lee D.S. (2017). AgNP and rhEGF-incorporating synergistic polyurethane foam as a dressing material for scar-free healing of diabetic wounds. RSC Adv..

[121] Singla R., Soni S., Patial V., Kulurkar P.M., Kumari A., S M., Padwad Y.S., Yadav S.K. (2017). Cytocompatible Anti-microbial Dressings of Syzygium cumini Cellulose Nanocrystals Decorated with Silver Nanoparticles Accelerate Acute and Diabetic Wound Healing. Sci. Rep..

[122] Salouti M., Ahangari A., Sezer A.D. (2014). Nanoparticle based drug delivery systems for treatment of infectious diseases. Application of Nanotechnology in Drug Delivery.

[123] Ling L.L., Schneider T., Peoples A.J., Spoering A.L., Engels I., Conlon B.P., Mueller A., Schäberle T.F., Hughes D.E., Epstein S. (2015). A new antibiotic kills pathogens without detectable resistance. Nature.

[124] Dai X., Guo Q., Zhao Y., Zhang P., Zhang T., Zhang X., Li C. (2016). Functional Silver Nanoparticle as a Benign Antimicrobial Agent That Eradicates Antibiotic-Resistant Bacteria and Promotes Wound Healing. ACS Appl. Mater. Interfaces.

[125] Brogden K.A., Guthmiller J.M., Taylor C.E. (2005). Human polymicrobial infections. Lancet.

[126] Thattaruparambil Raveendran N., Mohandas A., Ramachandran Menon R., Somasekharan Menon A., Biswas R., Jayakumar R. (2019). Ciprofloxacin- and Fluconazole-Containing Fibrin-Nanoparticle-Incorporated Chitosan Bandages for the Treatment of Polymicrobial Wound Infections. ACS Appl. Bio. Mater..

[127] Liang Y., Chen B., Li M., He J., Yin Z., Guo B. (2020). Injectable Antimicrobial Conductive Hydrogels for Wound Disinfection and Infectious Wound Healing. Biomacromolecules.

[128] He J., Liang Y., Shi M., Guo B. (2020). Anti-oxidant electroactive and antibacterial nanofibrous wound dressings based on poly (ε-caprolactone)/quaternized chitosan-graft-polyaniline for full-thickness skin wound healing. Chem. Eng. J..

[129] Tauler Riera P., Mooren F.C. (2012). Redox Status. Encyclopedia of Exercise Medicine in Health and Disease.

[130] Apel K., Hirt H. (2004). Reactive oxygen species: Metabolism, oxidative stress, and signal transduction. Annu. Rev. Plant Biol..

[131] Liemburg-Apers D.C., Willems P.H., Koopman W.J., Grefte S. (2015). Interactions between mitochondrial reactive oxygen species and cellular glucose metabolism. Arch. Toxicol..

[132] Das K., Roychoudhury A. (2014). Reactive oxygen species (ROS) and response of antioxidants as ROS-scavengers during environmental stress in plants. Front. Environ. Sci..

[133] Birben E., Sahiner U.M., Sackesen C., Erzurum S., Kalayci O. (2012). Oxidative stress and antioxidant defense. World Allergy Organ. J..

[134] Montezano A.C., Dulak-Lis M., Tsiropoulou S., Harvey A., Briones A.M., Touyz R.M. (2015). Oxidative stress and human hypertension: Vascular mechanisms, biomarkers, and novel therapies. Can. J. Cardiol..

[135] Giacco F., Brownlee M., Schmidt Ann M. (2010). Oxidative Stress and Diabetic Complications. Circ. Res..

[136] Wu H., Li F., Shao W., Gao J., Ling D. (2019). Promoting Angiogenesis in Oxidative Diabetic Wound Microenvironment Using a Nanozyme-Reinforced Self-Protecting Hydrogel. ACS Cent. Sci..

[137] Bairagi U., Mittal P., Singh J., Mishra B. (2018). Preparation, characterization, and in vivo evaluation of nano formulations of ferulic acid in diabetic wound healing. Drug Dev. Ind. Pharm..

[138] Huijberts M.S.P., Schaper N.C., Schalkwijk C.G. (2008). Advanced glycation end products and diabetic foot disease. Diabetes Metab. Res. Rev..

[139] Niu Y., Xie T., Ge K., Lin Y., Lu S. (2008). Effects of extracellular matrix glycosylation on proliferation and apoptosis of human dermal fibroblasts via the receptor for advanced glycosylated end products. Am. J. Dermatopathol..

[140] Peres G.B., Schor N., Michelacci Y.M. (2017). Impact of high glucose and AGEs on cultured kidney-derived cells. Effects on cell viability, lysosomal enzymes and effectors of cell signaling pathways. Biochimie.

[141] Goova M.T., Li J., Kislinger T., Qu W., Lu Y., Bucciarelli L.G., Nowygrod S., Wolf B.M., Caliste X., Yan S.F. (2001). Blockade of receptor for advanced glycation end-products restores effective wound healing in diabetic mice. Am. J. Pathol..

[142] Liang Y.J., Jian J.H., Liu Y.C., Juang S.J., Shyu K.G., Lai L.P., Wang B.W., Leu J.G. (2010). Advanced glycation end products-induced apoptosis attenuated by PPARdelta activation and epigallocatechin gallate through NF-kappaB pathway in human embryonic kidney cells and human mesangial cells. Diabetes Metab. Res. Rev..

[143] Lee S.J., Lee K.W. (2007). Protective effect of (-)-epigallocatechin gallate against advanced glycation endproducts-induced injury in neuronal cells. Biol. Pharm. Bull..

[144] Lateef H., Aslam M.N., Stevens M.J., Varani J. (2005). Pretreatment of diabetic rats with lipoic acid improves healing of subsequently-induced abrasion wounds. Arch. Dermatol. Res..

[145] Chen S.A., Chen H.M., Yao Y.D., Hung C.F., Tu C.S., Liang Y.J. (2012). Topical treatment with anti-oxidants and Au nanoparticles promote healing of diabetic wound through receptor for advance glycation end-products. Eur. J. Pharm. Sci..

[146] Huang Y.-H., Chen C.-Y., Chen P.-J., Tan S.-W., Chen C.-N., Chen H.-M., Tu C.-S., Liang Y.-J. (2014). Gas-injection of gold nanoparticles and anti-oxidants promotes diabetic wound healing. RSC Adv..

[147] Ponnanikajamideen M., Rajeshkumar S., Vanaja M., Annadurai G. (2019). In Vivo Type 2 Diabetes and Wound-Healing Effects of Antioxidant Gold Nanoparticles Synthesized Using the Insulin Plant Chamaecostus cuspidatus in Albino Rats. Can. J. Diabetes.

[148] Chen L., Remondetto G.E., Subirade M. (2006). Food protein-based materials as nutraceutical delivery systems. Trends Food Sci. Technol..

[149] Administration D. (2011). Guidance for industry considering whether an FDA-regulated product involves the application of nanotechnology. Biotechnol. Law Rep..

[150] Tyner K.M., Zou P., Yang X., Zhang H., Cruz C.N., Lee S.L. (2015). Product quality for nanomaterials: Current U.S. experience and perspective. Wiley Interdiscip. Rev. Nanomed. Nanobiotechnol..

[151] Sainz V., Conniot J., Matos A.I., Peres C., Zupančič E., Moura L., Silva L.C., Florindo H.F., Gaspar R.S. (2015). Regulatory aspects on nanomedicines. Biochem. Biophys. Res. Commun..

[152] Ventola C.L. (2017). Progress in nanomedicine: Approved and investigational nanodrugs. Pharm. Ther..

[153] Juillerat-Jeanneret L., Dusinska M., Fjellsbo L.M., Collins A.R., Handy R.D., Riediker M. (2015). Biological impact assessment of nanomaterial used in nanomedicine. Introduction to the NanoTEST project. Nanotoxicology.

[154] Anderson J.M. (2012). Biocompatibility. Polym. Sci. A Compr. Ref. 10 Vol. Set.

[155] Hussain S.M., Warheit D.B., Ng S.P., Comfort K.K., Grabinski C.M., Braydich-Stolle L.K. (2015). At the crossroads of nanotoxicology in vitro: Past achievements and current challenges. Toxicol. Sci..

[156] De Jong W.H., Hagens W.I., Krystek P., Burger M.C., Sips A.J.A.M., Geertsma R.E. (2008). Particle size-dependent organ distribution of gold nanoparticles after intravenous administration. Biomaterials.

[157] Keck C.M., Müller R.H. (2013). Nanotoxicological classification system (NCS)—A guide for the risk-benefit assessment of nanoparticulate drug delivery systems. Eur. J. Pharm. Biopharm..

[158] Han H. (2016). The effect of nanoparticle size on in vivo pharmacokinetics and cellular interaction. Nanomedicine.

[159] Layliev J., Wilson S., Warren S.M., Saadeh P.B. (2012). Improving Wound Healing with Topical Gene Therapy. Adv. Wound Care.

[160] Naderi N., Karponis D., Mosahebi A., Seifalian A.M. (2018). Nanoparticles in wound healing; from hope to promise, from promise to routine. Front. Biosci..

[161] Gunatillake P.A., Adhikari R. (2003). Biodegradable synthetic polymers for tissue engineering. Eur. Cell Mater..

[162] Li W., Tsen F., Sahu D., Bhatia A., Chen M., Multhoff G., Woodley D.T. (2013). Extracellular Hsp90 (eHsp90) as the actual target in clinical trials: Intentionally or unintentionally. Int. Rev. Cell Mol. Biol..

[163] Khvalevsky E.Z., Gabai R., Rachmut I.H., Horwitz E., Brunschwig Z., Orbach A., Shemi A., Golan T., Domb A.J., Yavin E. (2013). Mutant KRAS is a druggable target for pancreatic cancer. Proc. Natl. Acad. Sci. USA.

[164] Nethi S.K., Das S., Patra C.R., Mukherjee S. (2019). Recent advances in inorganic nanomaterials for wound-healing applications. Biomater. Sci..

